# Antagonistic mechanism of *Bacillus velezensis* HX0039 as a biocontrol agent against *Trichoderma virens*-induced “Sanghuang” green mold

**DOI:** 10.1128/aem.00005-25

**Published:** 2025-07-08

**Authors:** Ying Zhang, Wenheng Gao, Hui Zhang, Tao Sun, Huixiang Yang, Yan Liu, Xiaoyang Han, Dengke Yin, Weifang Xu

**Affiliations:** 1 Joint Research Center for Chinese Herbal Medicine of Anhui of IHM, Anhui University of Chinese Medicine 117843, Hefei, China; 2 Anhui Province Key Laboratory of Research and Development of Chinese Medicine, School of Pharmacy, Anhui University of Chinese Medicine 658051 , Hefei, China; 3 College of Life Science and Technology, China Pharmaceutical University 56651, Nanjing, China; 4 Institute of Sericulture Science and Technology Research, Chongqing, China; The Pennsylvania State University, University Park, Pennsylvania, USA

**Keywords:** "Sanghuang" mushroom, green mold, *Bacillus velezensis*, biocontrol, lipopeptide, genome mining

## Abstract

Green mold disease caused by *Trichoderma* spp. is one of the serious challenges in the production of “Sanghuang” mushroom (*Sanghuangporus vaninii*). Herein, we report the biocontrol efficacy and related mechanisms of *Bacillus velezensis* HX0039, an antagonistic strain previously isolated from soil for field cultivation of the “Sanghuang” mushroom. Strain HX0039 exhibited broad-spectrum activity against 17 fungal pathogens, especially *Trichoderma virens* SH4 (a highly pathogenic strain causing “Sanghuang” green mold), and increased the cell membrane permeability of *T. virens in vitro*. In the “Sanghuang” mode of green mold disease, HX0039 had no negative effects on the mycelial growth or fruiting body formation of mushrooms and exhibited excellent biocontrol efficacy. Genomic analysis revealed that HX0039 possesses multiple genes that contribute to antimicrobial compound production, and iturin A, fengycin, macrolactin A, surfactin, and bacillibactin were detected through UPLC-Q-Exactive-Orbitrap-MS. Among these metabolites, iturin A may play a role in combating *T. virens* SH4. Hemolytic tests, cell experiments, and mouse studies demonstrated that HX0039 is a biosafe strain. Our results will provide insights into the antifungal mechanisms of *B. velezensis* HX0039 and will facilitate the further application of this strain as a biocontrol agent against *T. virens*-induced “Sanghuang” green mold.

**IMPORTANCE:**

“Sanghuang” mushroom is a valuable edible and medicinal fungus. It provides numerous health benefits, including antitumor, anti-inflammatory, and immunity-enhancing properties. However, its growth is affected by green mold disease caused by *Trichoderma,* which severely hampers yield and quality. Conventional fungicide-based control methods have drawbacks like health and environmental risks, as well as the emergence of resistant pathogens. This study innovatively focuses on *Bacillus velezensis* HX0039, a strain proven to be safe for both “Sanghuang” mushrooms and mice. *In vivo* and *in vitro* experiments showed that HX0039 not only exhibits strong antifungal activity but also effectively prevents and controls the occurrence of “Sanghuang” green mold disease. Furthermore, the novelty of this research lies in its potential mechanism of action: HX0039 produces diverse metabolites like lipopeptides (especially iturin A), macrolactin A, and bacillibactin to exert its antagonistic activities. Therefore, this work demonstrates the great potential of *B. velezensis* HX0039 as an alternative to chemical fungicides in “Sanghuang” production.

## INTRODUCTION

The “Sanghuang” mushroom (*Sanghuangporus vaninii*), a basidiomycete fungus, is renowned for its edible and medicinal value. In China, the “Sanghuang” mushroom is also called “forest gold,” and it contains abundant polysaccharides, flavonoids, triterpenoids, and other bioactive substances that are beneficial for human health (1). As a traditional Chinese medicine, the “Sanghuang” mushroom is believed to have a variety of pharmacological properties, including antitumor, anti-inflammatory, hypoglycemic, autoimmunity-enhancing, and blood circulation-improving activities (2–4). Unfortunately, the productivity of the “Sanghuang” mushroom has been greatly threatened by green mold disease in different mushroom-planting zones. Analogous to other large-scale mushroom production methods, “Sanghuang” green mold disease is caused by *Trichoderma* species, such as *T. virens, T. harzianum*, and *T. aggressivum* (5, 6). These *Trichoderma* species generally contaminate spawn, culture substrates, and wood in mushroom cultivation farms, hindering the development of “Sanghuang” mycelia and fruiting bodies and resulting in a drastic reduction in mushroom production.

The prevention and control of green mold disease relies primarily on the maintenance of strict hygiene in mushroom production facilities and the application of fungicides, the latter of which are widely used in mushroom production for disease management (7). However, only a few fungicides have been approved for use in mushrooms, such as thiabendazole (a systemic benzimidazole) and imidazole (prochloraz) (8), and the long-term overuse of chemical agents might have adverse effects on human health and environmental safety. Moreover, the use of fungicides can lead to the emergence of drug-resistant pathogens, in turn leading to a decrease in the efficacy of fungicides and more severe consequences (9). Therefore, alternative management strategies for controlling “Sanghuang” green mold disease are urgently needed.

Recently, numerous studies have focused on the use of microbial inoculants for the biocontrol of crop diseases (10). *Bacillus* spp. are commonly found in nature and widely used as biocontrol agents because of their ability to antagonize phytopathogens and due to their potential applications in the development of stable spore-based commercial products (11). Species within the genus *Bacillus* are utilized not merely for the management of plant diseases, but also display significant potential for application in the realm of mushroom protection. In France, *Bacillus velezensis* QST713 has been widely used industrially for the protection of *Agaricus bisporus* from *Trichoderma*, which causes green mold disease (10, 12). Milijašević-Marčić et al. reported that *Bacillus subtilis* B-38 not only did not inhibit the growth of *A. bisporus* mycelia but also showed excellent biocontrol capacity against *Agaricus* green mold both *in vitro* and *in vivo* (13). Stanojevic et al. reported that the *Bacillus amyloliquefaciens* B-241 and *B. velezensis* QST713 strains exhibited similar and strong potential for application in the treatment of both compost green mold and dry bubble disease (14). These studies suggest that *Bacillus*-based biocontrol agents could serve as harmless alternatives to synthetic fungicides in mushroom production. However, there are few reports on the use of *Bacillus* spp. as biocontrol agents for controlling “Sanghuang” green mold caused by *Trichoderma* species.

With the application of *Bacillus* in agricultural production, more reports related to the biocontrol mechanisms of *Bacillus* have been published. Generally, the described mechanisms associated with their biocontrol effects include specific antagonistic effects, spatial and nutritional competition, induction of host systemic resistance, and stimulation of plant growth (11). One of the most important mechanisms by which *Bacillus* spp. directly inhibit pathogens is the synthesis of a great variety of secondary metabolites. Lipopeptide antibiotics produced by the non-ribosomal peptide pathway have attracted much attention in recent years (15). There are three main families of lipopeptides produced by *Bacillus*: the surfactin family, whose members have surfactant properties; the iturin family, whose members have outstanding antifungal activity; and the fengycin family, whose members also have antifungal activity (16). Notably, approximately 5%–10% of the whole genome of *Bacillus* spp. is dedicated to the synthesis of secondary metabolites (17). For example, *B. velezensis* FZB42 dedicates more than 10% of its genomic capacity to the synthesis of antimicrobial secondary metabolites (18, 19). In recent years, whole-genome sequencing has been increasingly shown to be useful for exploring the molecular mechanisms and antifungal genes of *Bacillus* species as potential biocontrol agents (20). Ultrahigh-performance liquid chromatography coupled with hybrid quadrupole-orbitrap mass spectrometry (UPLC-Q-Exactive-Orbitrap-MS) is a sensitive, reliable, rapid, comprehensive method that can also be used to analyze complex mixtures (21). The combination of whole-genome sequencing and liquid chromatography-mass spectrometry (LC-MS) technology has been shown to be an excellent approach for revealing the active components and mechanisms underlying biological action (22, 23).

Previously, we isolated the strain *Bacillus* sp. HX0039 from soil in the field cultivation of the “Sanghuang” mushroom, and an antagonistic assay revealed its excellent antifungal activity against *T. virens*, a major fungal pathogen responsible for green mold in “Sanghuang” mushroom (5). Our previous work revealed that the HX0039 strain is a promising biocontrol agent for “Sanghuang” green mold, but its biocontrol properties and related mechanisms remain to be explored. The present study further evaluated the biocontrol effects of strain HX0039 against *T. virens* SH4 through *in vivo* and *in vitro* experiments and explored its antifungal mechanism through whole-genome sequencing and metabolite analysis. Finally, its safety was evaluated to determine the safety of its use as a biocontrol agent.

## MATERIALS AND METHODS

### Microorganisms and culture conditions

The bacterial strain HX0039 was originally isolated from soil in the habitat of “Sanghuang” mushrooms sampled from the artificial Chinese medicine cultivation base in Jinzhai County, Anhui Province, China (31°46’28” N, 115°54’58” E) (5). Strain HX0039 was cultured in Luria-Bertani (LB) liquid medium (10 g/L peptone, 5 g/L yeast extract, and 10 g/L NaCl) with constant shaking at 180 rpm at 30°C for 18 h before use.

Seventeen pathogenic strains were used in this study. Two strains pathogenic to the “Sanghuang” mushroom (*T. virens* SH4 and *T. harzianum* SH2) and five putative pathogens to the “Sanghuang” mushroom (*Aspergillus* sp. SH15, *Fusarium* sp. SH17, *Fusarium* sp. SH18, *Penicillium* sp. SH19, and *Penicillium* sp. SH20) were collected in our laboratory (W. Xu, unpublished data); seven phytopathogens (*Fusarium oxysporum*, *Botrytis cinerea*, *Colletotrichum gloeosporioides*, *Fusarium solani*, *Rhizoctonia solani*, *Fusarium proliferatum*, and *Colletotrichum lagenarium*) were kindly provided by Professor Jie Xie of Southwest University (24–26); and the remaining three phytopathogens, *Cryptosporiopsis malicorticis*, *Colletotrichum gloeosporioides*, and *Glomerella cingulata*, were kindly provided by Professor Min Liao of Anhui Agricultural University (27). Among these fungi, only the *T. virens* SH4 strain was used in *in vitro* and *in vivo* tests of the biocontrol efficacy (BE) of the HX0039 strain, whereas the remaining 16 were used only in *in vitro* tests of the antagonistic activity of the HX0039 strain. All fungal isolates were cultured on potato dextrose agar (PDA; 200 g/L potato, 20 g/L dextrose, 15 g–20 g/L agar) at 25°C before use.

The “Sanghuang” culture bags used for *in vivo* experiments in this study were purchased from the Institute of Sericulture Science and Technology Research, Chongqing. The fungal species of the “Sanghuang” mushroom was identified as *Sanghuangporus vaninii* (28, 29).

### Antifungal activity of strain HX0039 *in vitro*

Dual-culture tests were used to examine the effects of HX0039 cell suspensions on the mycelial growth of 17 fungal pathogens (30). Briefly, one mycelial disk (5 mm) of each pathogenic isolate was placed in the center of a PDA plate, and then, the HX0039 bacterial culture in the logarithmic phase (10^8^ CFU/mL) was streaked on opposite sides of the same plate 3.0 cm from the center and incubated at 25°C for 2–5 days. Plates that were inoculated with a pathogen plug in the same zone in the absence of the HX0039 cell suspension were tested as a control. The mycelial inhibition rate (I) was calculated using the following formula: I (%) = [(C – T) / (C – C_0_)] × 100, where C represents the growth diameter of the fungal pathogen in the control, T represents the growth diameter of the pathogen in the dual-culture plate, and C_0_ represents the diameter of the test fungal agar disks (5.0 mm). All the experiments were carried out in triplicate.

The effects of the cell-free supernatant of HX0039 on *T. virens* SH4 and *T. harzianum* SH2 were examined by the method reported by Li et al. (31). Briefly, fresh HX0039 cultures were inoculated into LB medium and incubated at 30°C for 96 h at 180 rpm. The cultures were then centrifuged at 12,000 rpm for 30 min and filtered through a 0.22 µm biofilter to discard the cells, and the cell-free supernatant was thus obtained. The cell-free supernatant was mixed into the medium for PDA plates at a ratio of 1:8 to prepare plates containing HX0039 cell-free supernatant. SH4 or SH2 was inoculated into the middle of the above plates and cultured at 25℃ for 2 days. Plates that were inoculated with a pathogen plug in the same zone in the absence of the HX0039 cell-free supernatant were used as a control. The inhibition rate obtained by this method was the same as that obtained by the dual-culture method described above, and all the experiments were carried out in triplicate.

To analyze the thermal stability of the secondary metabolites of HX0039, aliquots of the cell suspension (or cell-free supernatant) were treated independently at 60, 80, or 100°C for 30 min and then subjected to bioassays as described in our previous work (32). Briefly, fresh *T. virens* SH4 agar disks (5.0 mm) were placed at the centers of PDA plates, and 100 µL of heat-treated HX0039 cell suspension (or cell-free supernatant) was added to the wells 3.0 cm from the center. LB medium and cell suspensions (or cell-free supernatants) that were not subjected to high temperatures served as two controls. The plates were incubated at 25°C for 2 days, after which the diameters (Di) of the fungal inhibition zones were measured. All the treatments were performed in triplicate.

### *In vivo* experiments

#### Preparation of the green mold inoculum and bacterial suspensions

Conidia from 5-day-old cultures of *T. virens* SH4 were flooded with 10 mL of sterilized distilled water (SDW) and Tween 20 (vol/vol 0.01%), followed by filtration through double layers of cheese cloth. The concentration of conidia used in the mushroom growth room as the inoculum was adjusted to 10^10^ conidia/mL. Fresh HX0039 cultures were inoculated into LB broth and incubated at 30°C for 24 h at 180 rpm. Then, the cultures were subsequently centrifuged at 12,000 rpm for 30 min, and the bacterial pellets were subsequently resuspended in SDW. The inoculum concentration was adjusted to 10^9^ CFU/mL using SDW.

#### Test in mushroom growth room

In this study, we selected the “Sanghuang” cultivation bags that had undergone a mycelium growth period of approximately 55 days and were about to enter the fruiting stage for *in vivo* experiments. The test culture bags were treated (HX0039 +) or not treated (HX0039 −) with strain HX0039 and inoculated (SH4 +) or not inoculated (SH4 −) with *T. virens* SH4. Four conditions were studied: control culture bag (group 1: HX0039 −, SH4 −), inoculated culture bag (group 2: HX0039 −, SH4 +), treated culture bag (group 3: HX0039 +, SH4 −), and inoculated and treated culture bag (group 4: HX0039 +, SH4 +). Six culture bags were used in each group, and the treatment process was presented as follows: the “Sanghuang” culture bags were prepared as described above, and after the “Sanghuang” mycelia had grown throughout the bags, the bags were cut open into rectangles (length × width: 3 cm × 1 cm). Finally, 1 mL of sterilized distilled water, the HX0039 suspension, the *T. virens* SH4 conidial suspension, and the *T. virens* SH4 conidial suspension + HX0039 suspension were injected into the opening of the culture bags of group 1, group 2, group 3, and group 4, respectively. All the culture bags were cultivated in a greenhouse at 25°C and 90% relative humidity with a 12 h photoperiod. Disease incidence in “Sanghuang” mushrooms was investigated approximately 1 week before harvest. The harvested mushrooms were weighed and divided into two groups based on visual observation, i.e., those with and those without symptoms of green mold disease. The disease incidence and BE of each treatment were calculated as follows: disease incidence (%) = the number of diseased “Sanghuang” mushrooms / the total number of “Sanghuang” mushrooms × 100; biocontrol efficacy (%) = (the disease incidence in group 2 − the disease incidence in group 4) / the disease incidence in group 2 × 100.

### Genome sequencing and identification of strain HX0039

#### Morphological, physiological, and biochemical characteristics

The morphological characteristics of strain HX0039 cultured on LB plates at 28°C for 24 h were recorded. Gram staining and spore staining were performed as described by Ou et al. (25) and then observed under an optical microscope. A series of biochemical tests, including glucose acid production, Voges-Proskauer, and hydrogen sulfide tests, were conducted using an HK-MID-66 kit (Guangdong Huankai Microorganism Co., Ltd.) according to methods described in the literature (33). The activities of protease, cellulase, and amylase from HX0039 were detected on agar plates containing skim milk, sodium carboxymethyl cellulose, and starch, respectively. Chrome-azurol S (CAS) medium was used to detect the ability of HX0039 to produce siderophores (34, 35).

#### Genome sequencing and assembly

The extraction of genomic DNA from strain HX0039 was performed as follows: a fresh single colony of strain HX0039 was picked and inoculated into LB broth, cultured in a 37°C shaker for 16 h, and centrifuged at 8,000 r/min for 30 min to collect the bacterial cells. Genomic DNA was extracted according to the instructions of the PrepMan Ultra Sample Preparation Reagent (Applied Biosystems, USA). After the samples passed the tests for purity (A260/280 was 1.8 ~ 2.0) and mass concentration (>100 ng/µL), they were sent to Wuhan GrandOmics Biosciences Co., Ltd. (Hubei, China) for whole-genome sequencing.

Single-molecule sequencing of DNA was performed using an Oxford Nanopore Technology GridION sequencer (36). After library construction, DNA libraries of a certain concentration and volume were added to the flow cell and transferred to the GridION sequencer for real-time single-molecule sequencing, obtaining the raw data, and performing genome assembly, correction, and optimization of the data after quality control. Average nucleotide identity (ANI) analysis was performed using the Pyani version 0.2.11 with the parameter -m ANIb set.

#### Gene prediction and functional annotation

Gene prediction and functional annotation were performed as follows: coding genes were predicted by Prodigal, and the complete protein-coding sequences (CDSs) were retained. After the extraction of the genomically encoded protein sequences, the proteins were annotated with InterProScan to extract the annotation information from the GO database; the protein sequences were aligned to the KEGG database with BLASTP, and the best hits with a coverage of more than 30% were retained as the annotation results. The encoded proteins were subsequently aligned to the COG database with rpsBLAST for annotation. The anti-SMASH 8.0.0 (https://antismash.secondarymetabolites.org/) was employed for the prediction and annotation of secondary metabolite biosynthetic gene clusters (BGCs). The ClusterBlast and KnownClusterBlast modules integrated into anti-SMASH 8.0.0 were also utilized to perform comparative gene cluster analyses based on the “Minimum Information about a Biosynthetic Gene Cluster” (MIBiG) data standard.

#### Molecular biological identification

Molecular biological identification of HX0039 was performed by constructing four phylogenetic trees based on three housekeeping genes (16S rDNA, *gyrA*, and *gyrB*) and single-copy orthologous genes related to *Bacillus*-related bacterial strains (26). The following primers were employed for amplifying three gene fragments (16S rRNA, *gyrA*, and *gyrB*): 27F (5′-AGAGTTTGATCCTGGCTCAG-3′) and 1492R (5′-GGTTACCTTGTTACGACTT-3′) targeting 16S rRNA; 42F (5′-CAGTCAGGAAATGCGTACGTCCTT-3′) and 1066R (5′-CAAGGTAATGCTCCAGGCATTGCT-3′) for *gyrA*; as well as UP-1S (5′-GAAGTCATCATGACCGTTCTGCA-3′) and UP-2Sr (5′-AGCAGGGTACGGATGTGCGAGCC-3′) for *gyrB* (37). Three phylogenetic trees based on the above single housekeeping gene were respectively constructed using the neighbor-joining method in MEGA software version 7.0 with 1,000 replicates of bootstrap values. Moreover, the 16S rRNA, *gyrA*, and *gyrB* gene sequences were submitted to GenBank under the accession numbers OP268577, OR859838, and OR859839, respectively.

In addition, the steps for constructing the phylogenetic tree based on single-copy orthologous genes were as follows: the complete genomes of 20 *Bacillus* strains with biocontrol functions were downloaded from NCBI GenBank and annotated using the Prokka software version 1.14.6; subsequently, the single-copy orthologous genes were identified using the OrthFinder software version 2.5.4 based on the obtained protein sequences; finally, the IQ-Tree software version 2.2.0.3 was employed to construct a phylogenetic tree using the maximum-likelihood method with 1,000 replicates of bootstrap values.

### Extraction, antifungal activity test, and molecular mass determination for HX0039 lipopeptides

#### Extraction and antifungal activity of HX0039 lipopeptides

Crude lipopeptide extracts (CLPs) were extracted after inoculation of strain HX0039 into Landy medium for 72 h according to methods described by Chen et al. (38). Briefly, the fermentation supernatant was collected by centrifugation, and the pH was adjusted to 2.0 via the addition of 6 mol/L HCl. After precipitation overnight at 4°C, the precipitate was collected by centrifugation and extracted with methanol at least three times. Then, the methanol was evaporated under vacuum to dryness; the final CLPs were dissolved in methanol, and 20 mg/mL and 10 mg/mL solutions were prepared. The antifungal activity of the CLPs was evaluated by a disk diffusion assay (39). Filter paper disks were soaked with 10 µL of CLPs and placed 2 cm from the periphery of a PDA plate containing a fungal disk in the center. A methanol-soaked disk was used as a control.

The minimum inhibitory concentrations (MICs) of HX0039 CLPs were determined in sterile 96-well microplates with a final volume of 100 µL in each microplate well. Twofold serial dilutions of the extracts were prepared in the microplate wells over the range of 7.1–454.4 μg/mL. Ten microliters of a mycelial suspension of *T. virens* SH4 was added to each test well. The plates were then incubated in an incubator at 25°C for 48 h. The MIC was defined as the lowest concentration of the extract at which the mycelia of *T. virens* SH4 did not show visible growth after inoculation. To determine the minimum fungicidal concentrations (MFCs), 10 µL of the cultures was inoculated into PDA media and incubated at 25°C for 48 h. The concentration at which there was no growth was considered the MFC, indicating that >99.9% of the original inoculum was killed. All assays were performed using two independent CLP batches (biological replicates), with triplicate technical measurements per batch to assess variability.

*T*. *virens* SH4 mycelia and conidia were treated with CLPs at the concentration of 1 × MIC. *T*. *virens* SH4 mycelia were picked and added to PDB with or without CLPs, incubated for 24 h, and observed under a light microscope. To test the effects of CLPs on conidial germination, CLPs (1 × MIC) were added to water agar (WA) medium at 50°C, after which the mixture was homogenized. A slide was dipped in WA medium, which was then allowed to solidify, after which a suspension of *T*. *virens* SH4 conidia was evenly coated on its surface. The conidia treated with or without CLPs were observed under a light microscope after 24 h.

#### Identification of HX0039 lipopeptides

HX0039 CLPs, obtained as described above, were dissolved in methanol and filtered through a 0.22 µm biofilter to discard the cells. LC-MS/MS analysis of the CLPs was performed on a 1290 UPLC system coupled with a Q-Exactive Plus Orbitrap mass spectrometer equipped with a heated electrospray ionization source (Thermo Fisher Scientific-CN, Orbitrap Exploris 120, Germany). CLPs were eluted using a two-component solvent system, in which solvent A was 0.1% formic acid in water and solvent B was 0.1% formic acid in acetonitrile. A total of 4 µL of sample was injected onto an ACQUITY UPLC BEH C18 column (1.7 µm, 2.1 mm × 50 mm, Waters), and gradient elution was performed as follows: 5% B from 0 to 2 min, 5%–95% B from 2 to 10 min, and 95% B from 10 to 15 min. The system was returned to the initial conditions of 5% from 15 to 18 min. The flow rate was 0.3 mL/min, and the column temperature was 30°C. The scanning mode was positive and negative ion mode switching, and the detection mode was full scanning/data-dependent secondary scanning (full MS/dd-MS2), with a scanning range of *m/z* 150–2,000. The full MS resolution was 60,000, the dd-MS2 resolution was 30,000, the electrospray voltage was 3.8 kV, the sheath gas pressure (N_2_ >99%) was 40 bar, the auxiliary gas pressure (N_2_ >99%) was 10 bar, the temperature of the ion transfer tube was 320°C, and the collision energy was 40 eV. The obtained raw files were imported into Xcalibur and Compound Discoverer 3.3 software to visualize and analyze the results. Total ion current chromatograms of HX0039 CLPs in positive and negative ion modes were visualized and saved using Xcalibur software. Subsequently, feature peak extraction was performed via Compound Discoverer 3.3, followed by simultaneous identification of characteristic peaks using both mzCloud and mzVault databases. The mass spectrometric fragmentation patterns of compounds were deduced from MS/MS fragment ions to determine molecular formulas and structures of HX0039 CLPs, with the compound qualitative analysis further validated by relevant literature (37, 40–44).

#### Preparation and identification of substances in the metabolites of strain HX0039 that may play a major role in antagonizing *T. virens*

To identify the key antifungal substances secreted by HX0039, the dual-culture method described in “Antifungal activity of strain HX0039 *in vitro*,” above, was employed to co-culture HX0039 with SH4. The experiment was divided into three groups: the HX0039 monoculture group, serving as the blank control; the SH4 monoculture group, serving as the experimental control; and the HX0039-SH4 dual-culture group, serving as the experimental group. After incubation, agar surrounding the colonies of each group was collected, pulverized, and extracted using methanol to isolate the metabolites. Subsequently, the chemical composition and differential metabolites among the groups were analyzed using high-performance liquid chromatography (HPLC) (Agilent 1260 Infinity II). Chromatographic separation was performed on an ACQUITY UPLC BEH C18 column (1.7 µm, 2.1 mm × 50 mm, Waters) with a mobile phase consisting of solvent A (water) and solvent B (acetonitrile). An isoelution gradient of 95:5 was applied, and the system was returned to the initial condition of 5% B from 18 to 22 min. The injection volume was 10 µL, with a flow rate of 1 mL/min and a column temperature maintained at 30°C. This method enabled the identification and preparation of compounds with significant differences in content between the groups. Finally, the prepared compounds were identified using the method described in “Identification of HX0039 lipopeptides,” above, to determine their potential antifungal activity.

#### Analysis of propidium iodide (PI) staining

The effects of HX0039 bacterial cell suspensions and CLPs on the *T. virens* SH4 cell membrane were examined using dual-culture and disk diffusion assays, respectively, as described above. Briefly, a coverslip was inserted between the HX0039 cells or disk and the SH4 mycelia, and the coverslip covered with SH4 mycelia was removed after 48 h of incubation. Then, the mycelia on the coverslips were stained with PI (50 µg/mL; Biosharp Technology Co., Ltd., Beijing, China) for 20 min, and the fluorescence intensity was observed under an inverted fluorescence microscope after the samples were rinsed with phosphate-buffered saline (PBS). Notably, in this study, the effects of CLPs at 4 × MIC and 8 × MIC on the bacterial cell membrane were detected, and a blank without drugs was used as a control.

### Biosafety testing

#### Assays of hemolysin production

A goat blood agar plate (Nanjing Quanlong Biology Technology Co., Ltd.) was used to detect the production of hemolysin. Strain HX0039 was inoculated on a blood agar plate and observed after 24 h. The method for determining hemolysis was as described by Li et al. (45).

#### Effects of HX0039 on the growth of cells and animals

The rat small intestinal crypt epithelial cell line IEC6 was obtained from Shanghai Zhongqiao Xinzhou Biotechnology Co., Ltd. The cells were cultured in Dulbecco’s modified Eagle medium (Shandong SparkJade Biotechnology Co., Ltd.) supplemented with 10% (vol/vol) fetal bovine serum (Anhui Kangyuan Biotechnology Co., Ltd.) and 1% (vol/vol) penicillin-streptomycin (Shandong SparkJade Biotechnology Co., Ltd.). The cells were incubated at 37°C in a 5% CO_2_ incubator. IEC6 cells were resuscitated, passaged, and distributed in 96-well plates, followed by the addition of medium containing CLPs, and cell viability was determined 24 h later using a CCK8 kit (Shandong SparkJade Biotechnology Co., Ltd.) according to the manufacturer’s instructions.

Kunming mice aged 6–8 weeks were obtained from Jiangsu Huachuang Xinnuo Pharmaceutical Technology Co., Ltd. A total of 36 mice were subjected to a 12 h light-dark cycle at a temperature of 25 ± 2°C and a humidity of 55%, and they were provided with *ad libitum* access to water and food. Prior to the commencement of the experiment, the mice were acclimated for a period of 1 week. Thirty-six Kunming mice were divided into six groups: the PBS group, the LB group, the cell-free supernatant group, and the *B. velezensis* HX0039 low-dose (1 × 10^6^ CFU/mL), medium-dose (1 × 10^8^ CFU/mL), and high-dose (1 × 10^10^ CFU/mL) groups. The gavage volume was 0.1 mL/10 g, and the mice were gavaged once. The status of the mice was observed daily, and the organ indices were calculated by dissecting and removing the organs after 7 days to assess toxicity. The organ index is calculated using the following formula: organ index = (organ weight / body weight) × 100% (46).

### Data analysis

The data were analyzed and visualized with GraphPad Prism 8. The data are expressed as the means ± standard deviations. A *t*-test was used to compare two groups, and one-way analysis of variance was used for multiple comparisons. Differences were considered statistically significant at *P* < 0.05.

## RESULTS

### Antifungal activity of strain HX0039 *in vitro*

**Fig 1:**
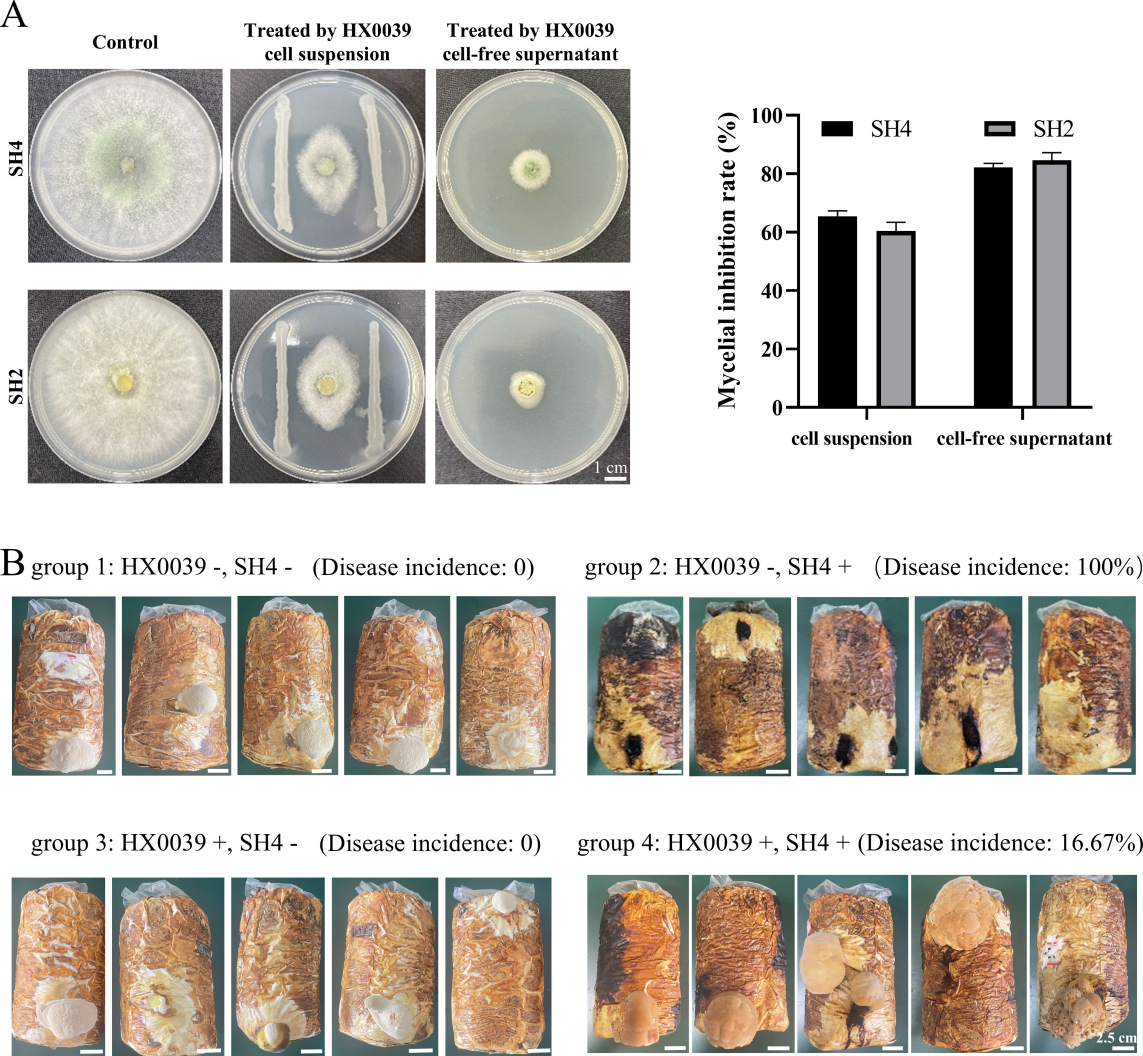
Antagonistic activities of the biocontrol bacterium HX0039 strain *in vitro* and *in vivo*. (**A**) Inhibitory effects of the HX0039 cell suspension and the HX0039 cell-free supernatant on the growth of *T. virens* SH4 and *T. harzianum* SH2. The left figure showed a control group where a mycelial disk of SH2 or SH4 was inoculated on normal PDA plate. In the middle figure, SH2 or SH4 was inoculated in the center of the PDA plate, and the HX0039 cell suspension (10⁸ CFU/mL) was streaked on both sides. In the right figure, SH2 or SH4 was inoculated in the center of the PDA plates containing the HX0039 cell-free supernatant, and the volume ratio of the HX0039 cell-free supernatant to the PDA medium was 1:8. Mycelial inhibition rate (I) was calculated using the following formula: I (%) = [(the growth diameter of the fungal pathogen in the control group − the growth diameter of the pathogen in the HX0039 treatment plate) / (the growth diameter of the fungal pathogen in the control group-the diameter of the tested fungal agar disc (5.0 mm)] × 100. (**B**) Biological control effects of strain HX0039 on “Sanghuang” green mold disease. Control culture bags (group 1: HX0039 −, SH4 −), inoculated culture bags (group 2: HX0039 −, SH4 +), treated culture bags (group 3: HX0039 +, SH4 −), inoculated and treated culture bags (group 4: HX0039 +, SH4 +). Note: The culture bags were treated (HX0039 +) or not treated (HX0039 −) with strain HX0039 and inoculated (SH4 +) or not inoculated (SH4 −) with *T. virens* SH4. The disease incidence of each treatment was calculated as follows: disease incidence (%) = the number of diseased “Sanghuang” mushrooms / the total number of “Sanghuang” mushrooms × 100.

Both the cell suspension and the cell-free supernatant of strain HX0039 were subjected to confrontation assays against *T. virens* SH4 and *T. harzianum* SH2. In the dual-culture test, strain HX0039 inhibited the growth of *T. virens* SH4 by 65.49% and that of *T. harzianum* SH2 by 60.39%. Compared with the growth of green molds on PDA medium, the growth of green molds on PDA medium containing HX0039 cell-free supernatant was obviously inhibited: the percentage inhibition of *T. virens* SH4 growth was 82.08%, and the percentage inhibition of *T. harzianum* SH2 growth was 84.58% (Fig. 1A). In addition, heat-treated cells and the cell-free supernatant of strain HX0039 also demonstrated potent antagonistic activity against *T. virens* SH4, suggesting that strain HX0039 cells and its antifungal metabolites are resistant to high temperature (Fig. S1).

To verify the robust antifungal activity of strain HX0039, 15 other pathogenic fungal species were selected for antifungal assays. The results demonstrated that strain HX0039 exhibited broad-spectrum antifungal activity. Specifically, it demonstrated a strong inhibitory effect not only on pathogenic fungi associated with “Sanghuang” mushroom but also on other prevalent plant pathogenic fungi (Fig. S2). These findings suggest that strain HX0039 displays a high level of antifungal activity *in vitro* and likely produces broad-spectrum antifungal substances.

### Biocontrol effects of strain HX0039 against *T. virens*-induced green mold in “Sanghuang” mushrooms

Mushroom growth bioassays were conducted to examine the efficacy of strain HX0039 in suppressing culture substrate-induced green mold disease in “Sanghuang” mushrooms. Four conditions were studied: natural control culture bags, which were neither treated with the antagonistic strain HX0039 nor inoculated with *T. virens* SH4 (the fungal pathogen of *S. vaninii*) (group 1: HX0039 −, SH4 −); culture bags inoculated with *T. virens* SH4 (group 2: HX0039 −, SH4 +); culture bags treated with the antagonistic strain HX0039 (group 3: HX0039 +, SH4 −); and culture bags treated with the antagonistic strain HX0039 and inoculated with *T. virens* SH4 (group 4: HX0039 +, SH4 +).

The growth status of “Sanghuang” mushrooms in the four *in vivo* treatment groups is shown in Fig. 1B. No symptoms of culture substrate-induced green mold appeared in group 1 or group 3. Inoculation of the culture with the pathogen *T. virens* SH4 (group 2) led to progressive invasion by this green mold at the opening of the culture bags, resulting in a black necrotic lesion in the substrate and a marked decrease in the development of *S. vaninii* mycelia and fruiting bodies relative to those under the other conditions. The highest incidence (100%) of culture substrate green mold was observed in group 2, and all “Sanghuang” mushrooms culture bags were infected by *T. virens* SH4. In contrast, strain HX0039 did not hinder mycelial growth or fruiting body formation in “Sanghuang” mushroom, regardless of the impact of the pathogen. Notably, strain HX0039 could effectively control the occurrence of “Sanghuang” green mold: the disease incidence in “Sanghuang” mushroom culture bags in group 4 was 16.67%, and its BE value was 83.33%.

### Species identification of strain HX0039

Strain HX0039 formed white colonies on LB plates, and the colonies had smooth surfaces and neat edges (Fig. S3A). In addition, HX0039 was characterized as a rod-shaped and gram-positive bacterium by Gram staining (Fig. S3B), and multiple budding spores were visible (Fig. S3C). Biochemical assays indicated that strain HX0039 could utilize glucose, sucrose, and lactose but not maltose or mannitol; the Voges-Proskauer reaction test was positive, and the hydrogen sulfide and urea tests were negative (Table 1). Translucent hydrolysis circles were formed around all colonies of strain HX0039 on the medium for cellulase, protease, and amylase assays, indicating that strain HX0039 could produce cellulase, protease, and amylase (Fig. S3D through F).

**TABLE 1:** Physiological and biochemical characteristics of strain HX0039^*a*^

Test item	Result	Test item	Result
Glucose	+	Simmonds citrate	+
Lactose	+	Hydrogen sulfide	−
Maltose	−	Urea	−
Mannitol	−	Semi-solid agar	−
Sucrose	+	Cellulase	+
Peptone water	−	Protease	+
Methylred test	−	Amylase	+
Voges-Proskauer	+		

^*a*^ + represents positive (growth or reaction), and − represents negative (no growth or no reaction).

**Fig 2:**
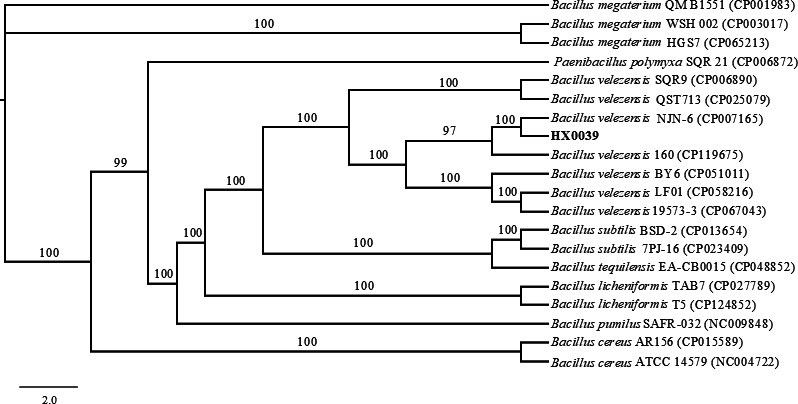
Phylogenetic tree of strain HX0039 based on single-copy orthologous genes of 20 *Bacillus*-related bacterial strains. The phylogenetic tree was constructed using the maximum-likelihood method in IQ-TREE software version 2.2.0.3, with 1,000 bootstrap replicates performed to assess branch support.

For the molecular biological identification of HX0039, we first constructed three phylogenetic trees based on the gene sequences of 16S rRNA, *gyrA*, and *gyrB*, respectively. The results showed that HX0039 forms a distinct clade with several *Bacillus velezensis* strains (16S rRNA gene: accession number OP985032; *gyrA* gene: accession numbers OP687993, OP687995, MW419812, and MT329072; *gyrB* gene: accession numbers MT437376, MT437378, MT437379, MT437380, and MT437381) (Fig. S4A through C). In addition, we calculated the ANI of HX0039 with several related strains and found that its ANI value with *Bacillus velezensis* was greater than 97% (Table S1). The phylogenetic tree of strain HX0039, which is based on single-copy orthologous genes, revealed that strain HX0039 is in the same minimal clade as *Bacillus velezensis* NJN-6 (CP007165) (Fig. 2). On the basis of its morphological, physiological, and biochemical characteristics and molecular identification, HX0039 was identified as *B. velezensis*.

### Genomic analyses of strain HX0039

### Genome assembly and annotation of strain HX0039

The complete genome of strain HX0039 consists of a 4,073,512 bp circular chromosome and one circular plasmid, pHX0039 (63,977 bp). The G+C contents of the chromosome and plasmid were 46.43% and 41.84%, respectively (Table S2 and Fig. S5A). A total of 4,002 complete CDSs were predicted, accounting for 88.54% of the whole-genome sequence, and the numbers of CDSs of the chromosome and plasmid were 3,921 and 81, respectively. The chromosome contains 82 tRNAs and 27 rRNAs (Table S2 and Fig. S5A). Functional analysis revealed that 3,037, 2,230, and 2,333 of the 4,002 complete CDSs could be annotated in the COG, KEGG, and GO databases, respectively. The relevant functional annotation analyses were detailed in Fig. S5B through D.

### Prediction of secondary metabolites related to biocontrol function

Anti-SMASH analysis predicted a total of 15 regions containing BGCs in the HX0039 genome. Among them, seven regions, 4, 5, 6, 11, 13, 14, and 15, exhibited no similarity with known gene clusters. One of them (region 8) exhibits low similarity with locillomycin biosynthesis gene cluster, and the remaining seven exhibit high similarity with known clusters (Table S3). Through in-depth analysis of the prediction results, we identified 13 individual BGCs whose sequences shared ≥80% gene similarity with entries in the MIBiG database. These BGCs are responsible for synthesizing diverse secondary metabolites, including non-ribosomal peptides (NRPs), polyketides, and ribosomally synthesized and post-translationally modified peptides (RiPPs). They represent the biosynthetic genes encoding for synthetases of fengycin (cluster 1), bacillomycin D (cluster 2), iturin (cluster 3), and plipastatin (cluster 4) in genomic region 1; bacillaene (cluster 5) in genomic region 2; macrolactin H (cluster 6) and macrolactin B (cluster 7) in genomic region 3; surfactin (cluster 8) in genomic region 7; bacilysin (cluster 9) in genomic region 9; amylocyclicin (cluster 10), bacillibactin (cluster 11), and paenibactin (cluster 12) in genomic region 10; and difficidin (cluster 13) in genomic region 12, respectively, (Table 2).

**TABLE 2:** Description of partial secondary metabolite BGCs in *B. velezensis* HX0039 genome^*a*^

Region	Cluster	Type	MIBiG accession	Metabolite	Similarity (%)
Region 1	1	NRPS	BGC0001095	Fengycin	94
	2	NRPS, polyketide	BGC0001090	Bacillomycin D	86
	3	NRPS, polyketide	BGC0001098	Iturin	86
	4	NRPS	BGC0000407	Plipastatin	83
Region 2	5	Polyketide, NRPS	BGC0001089	Bacillaene	89
Region 3	6	Polyketide	BGC0000181	Macrolactin H	91
	7	Polyketide	BGC0001383	Macrolactin B	91
Region 7	8	NRPS	BGC0000433	Surfactin	82
Region 9	9	Other	BGC0000888	Bacilysin	98
Region 10	10	RiPPS	BGC0000616	Amylocyclicin	95
	11	NRPS	BGC0001185	Bacillibactin	93
	12	NRPS	BGC0000401	Paenibactin	81
Region 12	13	Polyketide	BGC0000176	Difficidin	91

^*a*^ Genome analysis was performed using antibiotic and secondary metabolites analysis shell (anti-SMASH) and the MIBiG database. The above-listed BGCs refer to gene clusters with ≥80% gene similarity to known antibiotic gene clusters in the MIBiG database. NRPS, non-ribosomal peptide synthetase.

**Fig 3:**
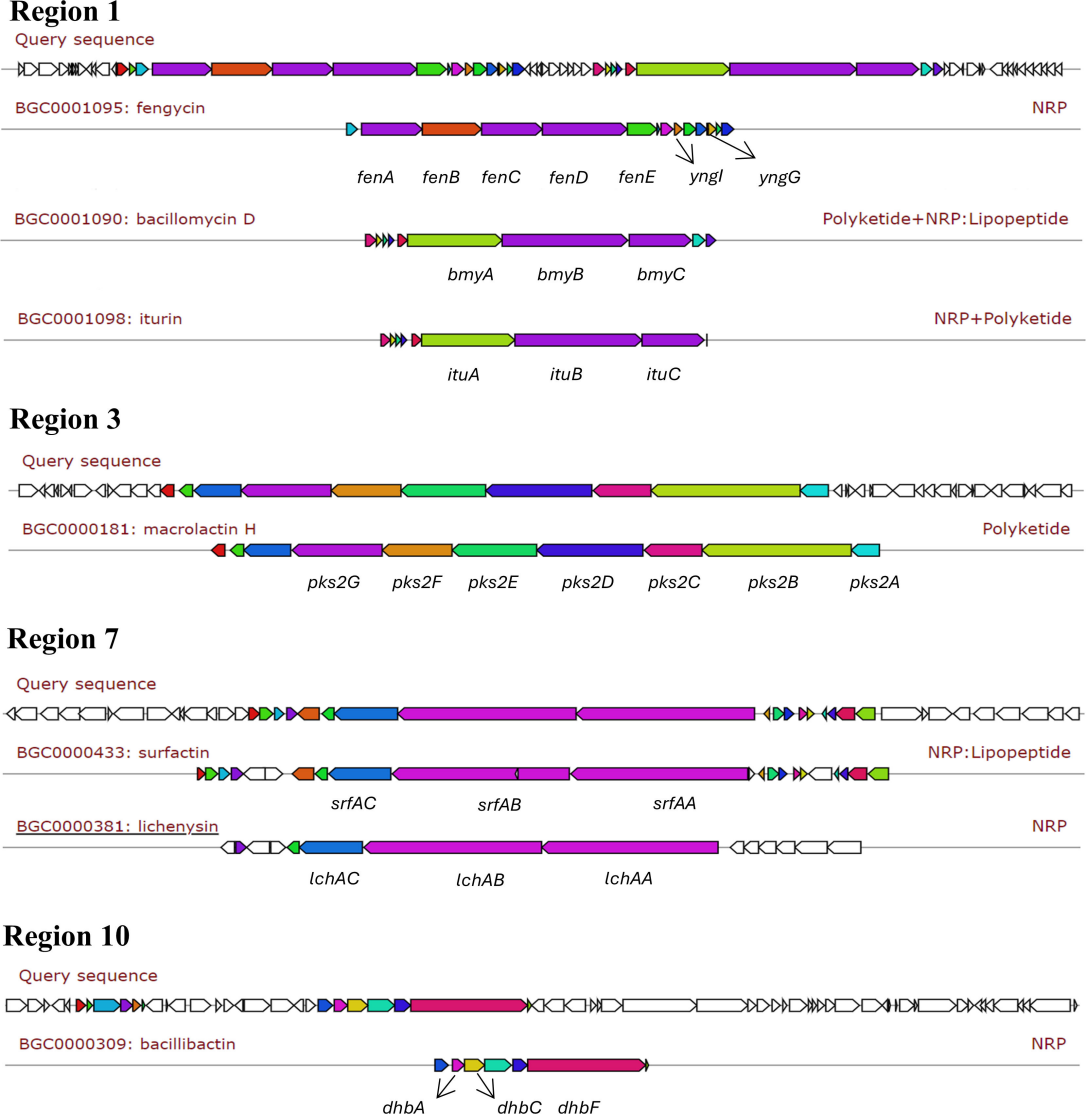
Comparative analysis of BGCs for query sequences in regions 1, 3, 7, and 10 of *B. velezensis* HX0039 genome with MIBiG database clusters at a ≥80% similarity threshold.

Identifications were made of the main biosynthetic genes, additional biosynthetic genes, regulatory genes, and other genes (Fig. 3 and Table S4). Furthermore, the fengycin gene cluster includes the core genes involved in biosynthesis, such as *yngG, yngI, fenA, fenB, fenC, fenD,* and *fenE*. Among the essential biosynthetic genes in the bacillomycin D gene cluster are *bmyA*, *bmyB,* and *bmyC,* and the iturin gene cluster’s core biosynthesis genes include *ituA*, *ituB,* and *ituC*. The core biosynthetic genes comprise the macrolactin H gene cluster (*pks2A*, *pks2B*, *pks2C*, *pks2D*, *pks2E*, *pks2F*, and *pks2G*). A number of other genes involved in surfactin biosynthesis (*srfAA, srfAB,* and *srfAC*) and bacillibactin biosynthesis (*dhbA, dhbC,* and *dhbF*) were found in the HX0039 genome. From the above anti-SMASH prediction results, we identified that the HX0039 genome harbors genes encoding lipopeptide antibiotics synthesized via NRPSs, including fengycin, bacillomycin D, iturin, and surfactin. Therefore, we hypothesized that the antifungal activity of strain HX0039 may be related to the secretion of lipopeptides, which prompted further investigation.

### Antifungal activity of the crude lipopeptide extracts

**Fig 4:**
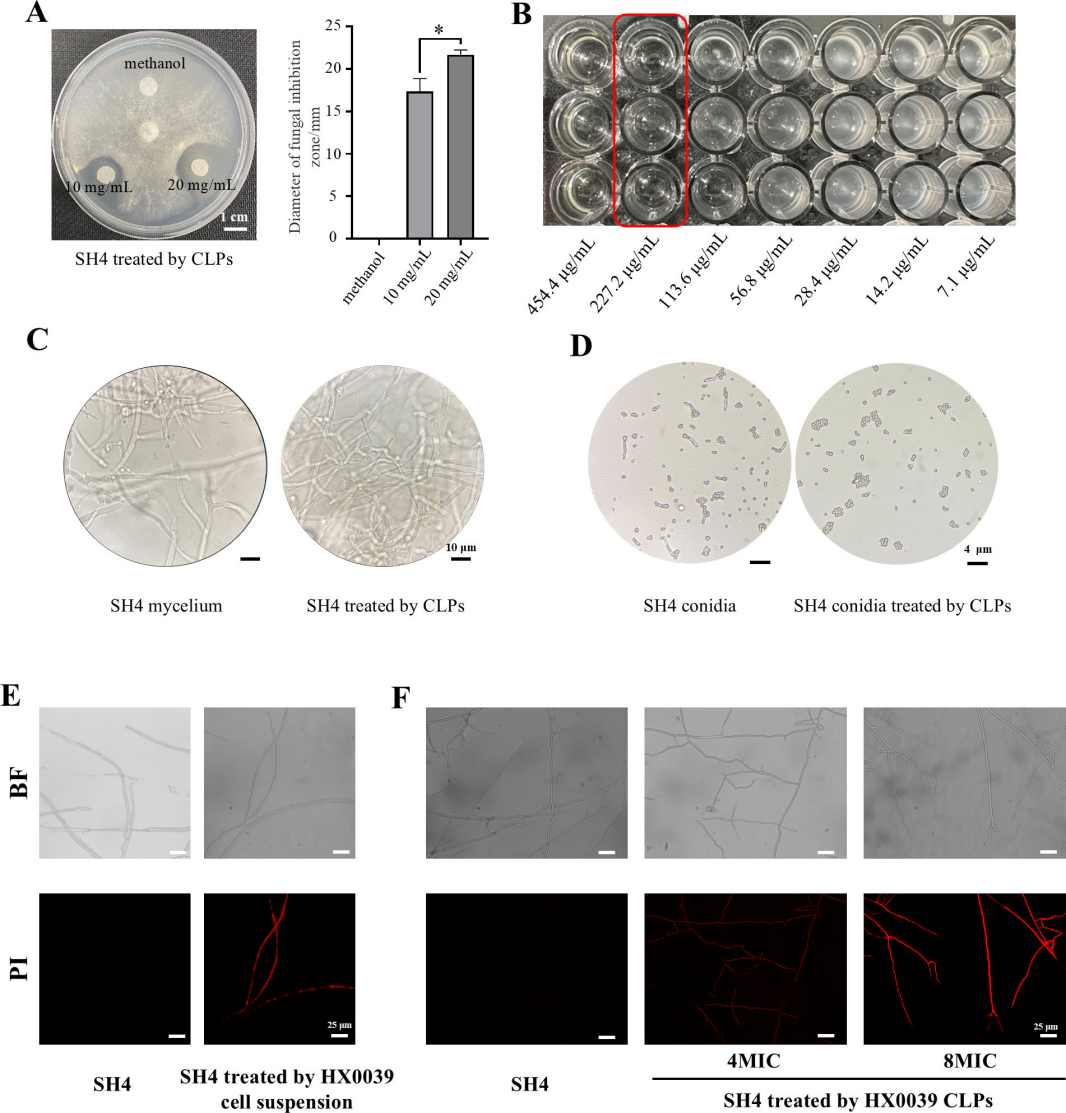
Antifungal activity of lipopeptides *B. velezensis* HX0039 fermentation supernatant. (**A**) Antagonistic effects in the disk diffusion assay. (**B**) Antagonistic effects in the microdilution assay. The numbers below the 96-well plate represent the concentrations of lipopeptide (LP) serial microdilutions. (**C**) Growth characteristics of *T. virens* mycelia treated with CLPs. (**D**) Effects of CLPs on the conidial germination of *T. virens*. (**E**) Effects of the cell suspension of strain HX0039 on the cell membrane of *T. virens* mycelia. (**F**) Effects of the HX0039 CLPs on the cell membrane of *T. virens* mycelia. BF = bright field.

Given that the HX0039 strain contains multiple genes encoding lipopeptide antibiotics, its CLPs were extracted and evaluated for their antagonistic effect on the growth of green mold *in vitro*. The hyphal growth was analyzed with different concentrations of HX0039 CLPs (Fig. 4). The mycelial diameter of *T. virens* SH4 significantly decreased with increasing concentrations of HX0039 CLPs on PDA plates; the diameter of the fungal inhibition zone at a concentration of 10 mg/mL was 17.33 ± 1.53 mm, whereas that at 20 mg/mL was 21.67 ± 0.58 mm (Fig. 4A). Microdilution assays revealed that both the MIC and MFC of HX0039 CLPs were 227.3 µg/mL (Fig. 4B). The experimental results of two separate batches of CLP in this study were consistent. In addition, the inhibition of *T. virens* SH4 growth by HX0039 CLPs *in vitro* was further detected by microscopic examination. The results showed that healthy-looking hyphae of *T. virens* SH4 not treated with HX0039 CLPs were regular in shape and could form abundant conidia, whereas deformed hyphae and fewer conidia were found in the hyphae of the pathogen co-cultured with the HX0039 CLPs (Fig. 4C). In addition to inhibiting hyphal growth and conidial emission, HX0039 CLPs negatively affected conidial germination (Fig. 4D). These results indicated that HX0039 CLPs exhibited antifungal activity.

### Effect of crude lipopeptide extracts from strain HX0039 on the *T. virens* SH4 cell membrane

In this study, PI staining assay was used to detect the effects of HX0038 cell suspension and CLPs on the *T. virens* SH4 cell membranes. As shown in Fig. 4, strong fluorescence was detected in *T. virens* SH4 mycelia co-cultured with strain HX0039, whereas there was almost no fluorescence when *T. virens* SH4 was cultured alone (Fig. 4E). In addition, with increasing concentrations of CLPs, the red fluorescence intensity gradually increased, whereas in the control, *T. virens* SH4 mycelia were not stained with PI (Fig. 4F). These results indicated that the HX0039 strain could destroy the integrity of the *T. virens* SH4 mycelial cell membrane, resulting in increased membrane permeability.

### Crude lipopeptide extract identification by UPLC-Q-Exactive-Orbitrap-MS

**Fig 5:**
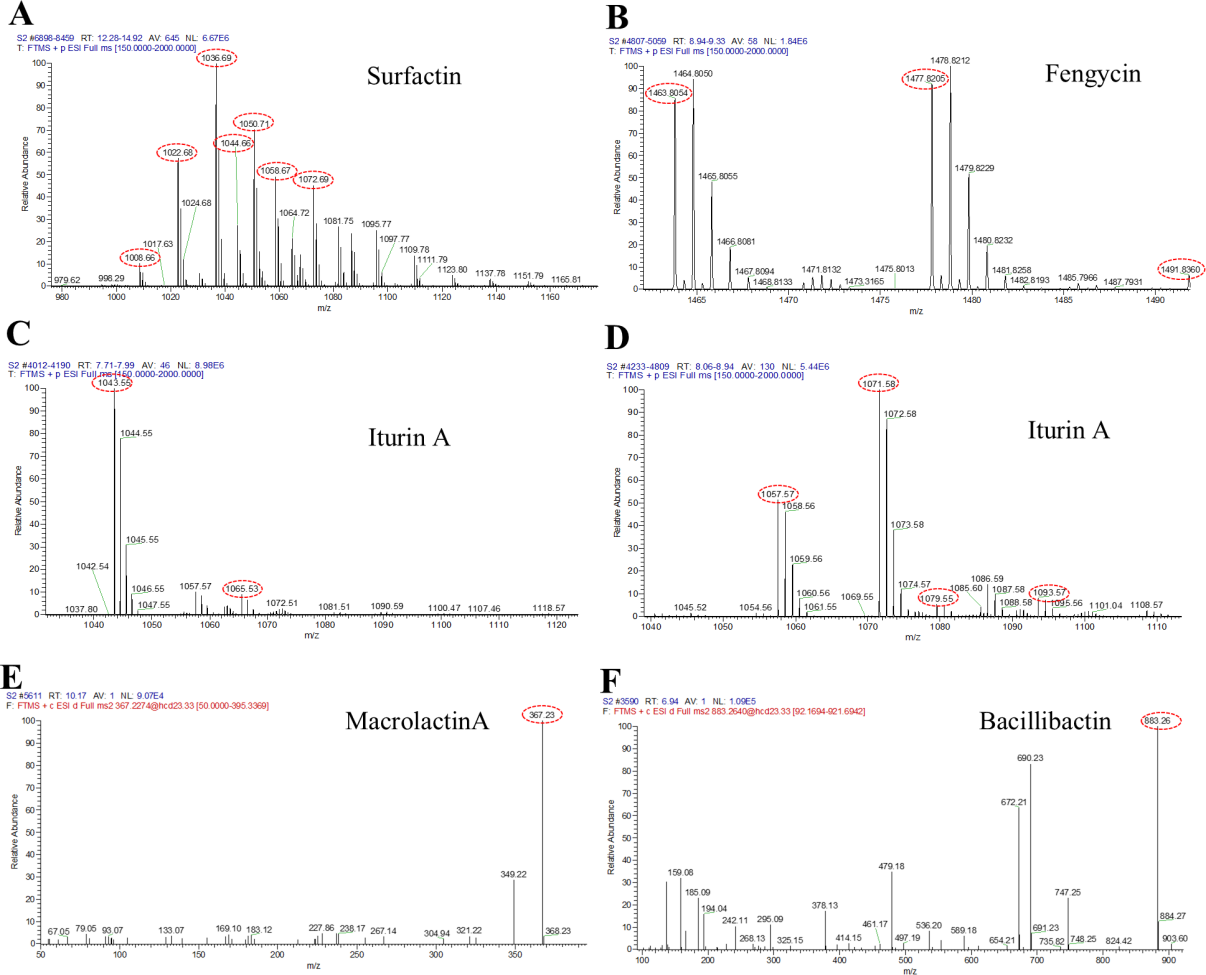
CLPs of strain HX0039 identified by UPLC-Q-Exactive-Orbitrap-MS. (**A**) Surfactin, (**B**) fengycin, (**C and D**) iturin A, (**E**) macrolactin A, (**F**) bacillibactin.

UPLC-Q-Exactive-Orbitrap-MS was used to identify the HX0039 CLPs. The results revealed that the CLPs contained several fractions of antifungal substances (Fig. 5 and Table 3). Surfactin was identified on the basis of mass spectrometry ion peaks at 1,008.66 *m/z*, 1,022.68 *m/z*, 1,036.69 m/z, 1,044.66 *m/z*, 1,050.71 *m/z*, 1,058.67 *m/z*, and 1,072.69 m/z (Fig. 5A) (37, 40). Fengycin was identified on the basis of the mass spectrometry ion peaks at 1,463.8504 *m/z*, 1,477.8205 *m/z*, and 1,491.8360 *m/z* (Fig. 5B) (40). Iturin A was identified on the basis of mass spectrometry ion peaks at 1,043.55 *m/z*, 1,057.57 *m/z*, 1,071.58 *m/z*, 1,065.53 *m/z*, 1,079.55, and 1,093.56 *m/z* (Fig. 5C and D) (41, 42). Macrolactin A and bacillibactin were identified based on the ion peaks at 367.23 *m/z* and 883.26 *m/z*, respectively (Fig. 5E and F) (43, 44). Notably, the presence of bacillibactin was verified by the transparent circles formed by *B. velezensis* HX0039 on a CAS plate (Fig. S4G).

**TABLE 3:** Assignments of major *m/z* peaks observed in the mass spectra of lipopeptides from strain HX0039

Mass peak (*m/z*)	Assignment	Reference
1,008.66	Surfactin A C13 [M + H]^+^ Surfactin B C14 [M + H]^+^	(37)
1,022.68	Surfactin A C14 [M + H]^+^ Surfactin B C15 [M + H]^+^	(37)
1,036.69	Surfactin A C15 [M + H]^+^	(37)
1,044.66	Surfactin A C14 [M + Na]^+^ Surfactin B C15 [M + Na]^+^	(40)
1,050.71	Surfactin A C16 [M + H]^+^	(40)
1,058.67	Surfactin A C15 [M + Na]^+^	(40)
1,072.69	Surfactin A C16 [M + Na]^+^	(40)
1,463.81	Fengycin A C16 [M + H]^+^	(40)
1,477.82	Fengycin A C17[M + H]^+^	(40)
1,491.84	Fengycin A C18 [M + H]^+^	(40)
1,043.55	Iturin A C14 [M + H]^+^	(41)
1,057.57	Iturin A C15 [M + H]^+^	(41)
1,071.58	Iturin A C16 [M + H]^+^	(41)
1,065.53	Iturin A C14 [M + Na]^+^	(41)
1,079.55	Iturin A C15 [M + Na]^+^	(41)
1,093.56	Iturin A C16 [M + Na]^+^	(42)
367.23	Macrolactin A [M-2H_2_O + H]^+^	(43)
883.26	Bacillibactin [M + H]^+^	(44)

### Detection of substances in the metabolites of strain HX0039 that may play a major role in antagonizing *T. virens*

**Fig 6:**
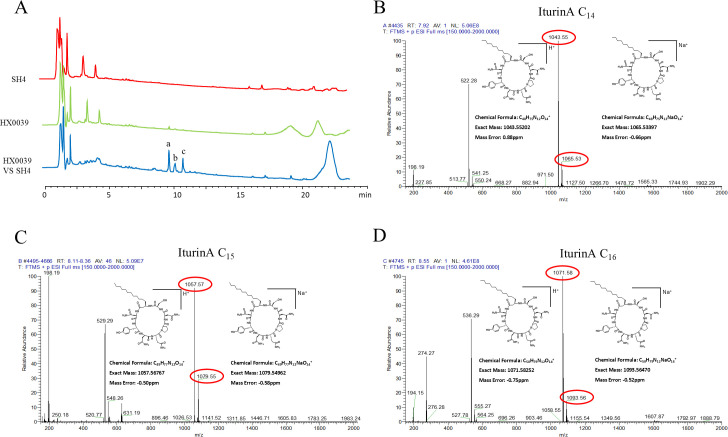
Preparation by HPLC and identification by UPLC-Q-Exactive-Orbitrap-MS of metabolites. (**A**) Chromatograms of metabolites in the three groups. (**B**) Mass spectra and ion peak structure of component A: iturin A C_14_. (**C**) Mass spectra ion peak structure of component B: iturin A C_15_. (**D**) Mass spectra ion peak structure of component C: iturin A C_16_.

To identify metabolites responsible for the antagonistic activity of HX0039 against *T. virens* SH4, HPLC analysis revealed distinct differences in metabolite profiles between the dual-culture and monoculture groups. Notably, three compounds (a, b, and c) of dual culture showed significant increases in concentration with retention times between 9 and 11 min (Fig. 6A). High-resolution liquid chromatography-mass spectrometry (UPLC-Q-Exactive-Orbitrap-MS) analysis of compound A revealed a [M  +  H]^+^ ion at *m*/*z* 1,043.55 and a [M  +  Na]^+^ ion at *m*/*z* 1,065.53, which is consistent with iturin A C14 (Fig. 6B). Similarly, compound B showed a [M  +  H]^+^ ion at *m*/*z* 1,057.57 and a [M  +  Na]^+^ ion at *m*/*z* 1,079.53, identifying it as iturin A C15 (Fig. 6C). Compound C was identified as iturin A C16 based on the [M  +  H]^+^ ion at *m*/*z* 1,071.58 and the [M  +  Na]^+^ ion at *m*/*z* 1,093.56 (Fig. 6D). The molecular weight differences among the three compounds corresponded to multiples of the methylene group (CH_₂_), indicating that compounds A, B, and C are iturin A homologs with varying fatty acid chain lengths (43, 44). The above results indicated that strain SH4 induces the production of iturin A analogs in strain HX0039. These compounds are likely the key contributors to the antagonistic activity of HX0039 against SH4.

### Safety of strain HX0039

**Fig 7:**
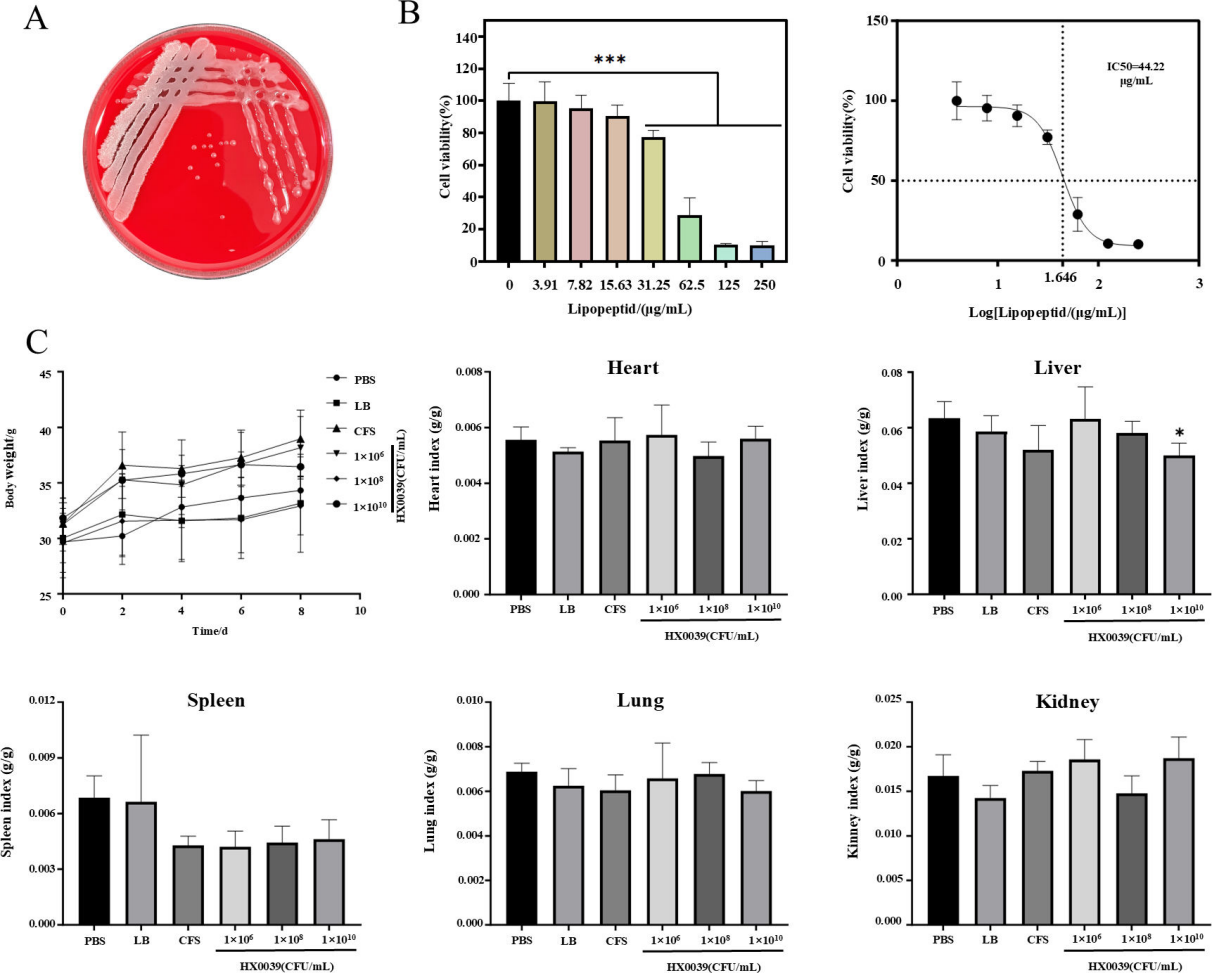
Safety tests of strain HX0039. (**A**) Growth status of strain HX0039 on a blood agar plate. (**B**) Effects of CLPs on the viability of IEC6 cells. (**C**) Body weight changes and organ indices of the mice. The organ index was calculated using the following formula: organ index = (organ weight / body weight) × 100%.

A hemolysis test revealed no hemolysis around the colony, indicating that the HX0039 strain did not produce hemolysin (Fig. 7A). When IEC6 cells were treated with CLPs, there was a significant difference in cell viability compared with that of the control group at a CLP extract concentration of 31.25 µg/mL, and the half maximal inhibitory concentration (IC50) of the CLPs in IEC6 cells was 44.22 µg/mL (Fig. 7B).

To ensure the safety of using the HX0039 strain for biocontrol of “Sanghuang” green mold disease in the field, a mouse infection model was established. Within 7 days after treatment, all the groups of mice exhibited normal body conditions, behavior, etc., and the weight of the mice in each group increased steadily. After 7 days, the heart, liver, spleen, lung, and kidney were dissected to calculate the organ indices, which were normal in all groups compared with those in the PBS group, except for the liver coefficient of the mice inoculated with 1 × 10^10^ CFU/mL, which was lower than that in the PBS group (Fig. 7C). These results indicated that HX0039 and its metabolites have favorable biosafety profiles for mammals.

## DISCUSSION

With the decreasing number of wild “Sanghuang” mushrooms, the scale of cultured “Sanghuang” production needs to be expanded. Recent studies have shown that cultured “Sanghuang” can be used as an alternative to the wild “Sanghuang” used in nutraceutical medicine (47). Unfortunately, green mold disease caused by *Trichoderma* species is a major problem in the production of “Sanghuang” mushrooms, as it not only reduces the production of “Sanghuang” mushrooms but also affects their quality. Compared with chemical control, biological control is considered a promising and sustainable alternative method due to the rarity of environmental contamination and low health risk (48). Therefore, research on the biocontrol of “Sanghuang” green mold is highly important for the sustainable development of “Sanghuang” production. However, overall, there has been less research on the biocontrol of fungal diseases of mushrooms than on plant fungal diseases. Although scientific and technological researchers have achieved promising results in the use of beneficial microorganisms (such as biocontrol *Bacillus* species and *Streptomyces* species) for the prevention and control of green mold disease in edible and medicinal mushrooms such as *Agaricus bisporus*, research on the indoor activity of most of these strains is still in the preliminary stage, and there are very few biocontrol bacteria that can be used for the prevention and control of green mold in edible and medicinal mushrooms in actual agricultural production (10, 12, 13, 49). In the present study, the efficacy of this strain HX0039 as a biocontrol agent for controlling “Sanghuang” green mold was studied through *in vitro* antagonistic activity and *in vivo* mushroom inoculation assays. In *in vitro* experiments, strain HX0039 not only exhibited high antagonistic activity against *T. virens* SH4, but also exhibited antagonistic activity against the other 16 pathogenic fungi (Fig. 1 and Fig. S2). In *in vivo* experiments test, *T. virens* SH4 caused the surface of the mushrooms to become black, hindering “Sanghuang” mushroom growth. The highest incidence of green mold (100%) was observed when “Sanghuang” mushroom culture bags were inoculated with only *T. virens* SH4. After treatment with strain HX0039, no inhibitory effect on the growth of “Sanghuang” fruiting bodies was observed; the disease incidence decreased to 16.67%, and the BE value was 83.33%. Overall, strain HX0039 has great potential as a biocontrol agent because of its ability to inhibit *Trichoderma* species growth and its broad-spectrum antifungal activity.

*Bacillus* species are the most important group of beneficial microorganisms with biocontrol effects. *Bacillus* species are a large and heterogeneous collection of aerobic or facultatively anaerobic, rod-shaped, endospore-forming bacteria that are widely distributed throughout the environment (50). *Bacillus* strains have attracted increasing attention due to their ability to produce durable and resilient endospores and antibiotics, which have the potential to be used as biocontrol agents (48). The populations of *Bacillus* species are extremely diverse. *B. velezensis* (strain CR-502T and strain CR-14b) was first isolated from environmental samples taken from the mouth of the Vélez River at Torredelmar in the province of Málaga, Spain (51). In recent years, *B. velezensis* has been widely studied as a bioprophylactic agent to control plant pathogens (52), but its application in disease control of edible and medicinal mushrooms is rare, and the use of this species to prevent and control green mold in “Sanghuang” has not been reported. In this study, we screened the biocontrol bacterium HX0039 from the soil of a “Sanghuang” cultivation base and identified it as the biocontrol bacterium *B. velezensis* based on morphological, physiological, and biochemical characteristics and phylogenetic tree analysis. As natural inhabitants of the human gut, many strains of *Lactobacillus* spp. and *Bifidobacterium* spp. have an established history of safe use in dietary supplements and foods such as yogurt, kefir, and cheese. Although *Bacillus* spp. have traditionally been described as soil-borne bacteria, they are also present in the naturally occurring human gut microbiota and, albeit less frequently, in commercially available probiotic products. Collectively, these data show that *Bacillus* spp. occur in the human gut in large enough numbers to be resident gut commensal bacterial species (53–55). The indirect introduction of *Bacillus* species to the food supply has occurred for decades through their use in animal production as feed additives (e.g., *B. subtilis*) without evidence of untoward effects on humans (56). The safe use of *Bacillus* strains is supported not only in healthy adults but also in pediatric populations (56, 57). However, not all strains of a particular species should be assumed to be safe for use in dietary supplements and food despite species inclusion on published lists of organisms generally presumed to be safe (58). In the present study, hemolytic, cellular, and mouse toxicity tests confirmed that the biocontrol agent not only did not affect the growth of “Sanghuang” mycelia or the formation of fruiting bodies, but also was nonhemolytic and did not have any significant negative effects on cellular or mouse growth. This is similar to the excellent safety profile reported for *Bacillus* (58). The results of the safety assessment demonstrated that *B. velezensis* HX0039 exhibited no adverse effects on mice and showed significant potential for further development as a biocontrol agent against the green mold disease of “Sanghuang” mushroom. However, its biosafety necessitates comprehensive evaluation through additional tests, including antibiotic resistance analysis and genotoxicity testing.

*Bacillus* strains exert their biocontrol effects predominantly through inhibiting the growth of plant pathogens, inducing systemic resistance in plants, and competing with plant pathogens for ecological niches (59). They are known to produce a variety of antagonistic compounds with different structures, and 5% to 10% of the genome is used for the biosynthesis of secondary metabolites (17). The most important bioactive molecules of the genus *Bacillus* are non-ribosomally synthesized peptides and lipopeptides, polyketide compounds, bacteriocins, and siderophores. Lipopeptides from *Bacillus* have a very complex biosynthetic mechanism catalyzed by NRPSs, which are large enzyme complexes with a modular structure in which each module is responsible for the synthesis of a specific amino acid. In general, they have a broad spectrum of antagonistic activities against plant pathogenic bacteria, fungi, and viruses. The most important molecules in this class of compounds are the cyclic lipopeptides of the surfactin, iturin, and fengycin families, which affect target cells at the membrane level (59).

Iturin A is a promising cyclic lipopeptide with multiple applications. Among its family members, iturin A exhibits structural differences while maintaining a shared cyclic peptide structure composed of seven α-amino acids and one β-amino acid. Iturin A, an efficient lipopeptide in the iturin family, possesses remarkable antifungal properties and minimal toxicity, thus being a promising candidate for the development of biopesticides and the treatment of fungal diseases (60). As a powerful biosurfactant, surfactin can interact with lipid components in biological membranes to disrupt fungal cell membranes, which is one of the main antimicrobial mechanisms. Surfactin can also disrupt nonmembrane substances such as proteins and nucleic acids to cause cell death (61). Similarly, fengycin is one of the most important compounds among the lipopeptides produced by *Bacillus* species, and its antimicrobial mechanism involves disruption of the cell membrane to allow leakage of the contents (62). Macrolactins A–Z constitute a family of macrolide antibiotics produced mainly by *Bacillus* spp. with antimicrobial and antitumor effects, among others (63). Iron is essential for the growth of all organisms, and most organisms rely on iron as a cofactor for important biochemical processes, including oxygen binding, electron transfer, and catalysis (64). *Bacillus* species secrete high concentrations of bacillibactin, produced by the non-ribosomal peptide pathway, to compete for Fe^3+^ binding with pathogenic fungi in the environment, which can promote their own reproduction and growth while causing the death of pathogenic fungi due to iron deficiency (19). In this study, we found that lipopeptides extracted from the fermentation broth significantly inhibited the mycelial growth and conidial germination of the pathogenic fungus *T. virens* and damaged its cell membrane, and we identified the above compounds in the extract; these phenomena are likely related to the abovementioned compounds. We also realized that when HX0039 was co-cultured with SH4, the types of substances produced by HX0039 might be altered. Further experimental analysis confirmed our speculation that more iturin A was produced by HX0039 during the cocultivation. Therefore, we hypothesized that iturin A is highly likely to be the antibiotic substance against *T. virens*. However, if further confirmation is necessary, it will be required to conduct further verification and analysis from aspects such as gene expression, which will be the goal of our next research stage.

*Bacillus* species can act as fungal inhibitors not only through the production of lipopeptides but also through the production of various extracellular enzymes and protein-like substances. We investigated the various types of antimicrobial substances that may be produced by strain HX0039. We inoculated HX0039 on various extracellular enzyme assay media and showed that HX0039 could produce three extracellular enzymes during growth: cellulase, amylase, and protease. Cellulose is an important component of the fungal cell wall, and the cellulase produced by HX0039 inhibits the ability of the fungus to produce cellulose, impeding the synthesis of the fungal cell walls as well as destroying them (65). Glycoproteins and ribosomal proteins in the cell wall of fungal pathogens are inhibited by proteases produced by strain HX0039 (66). The amylase produced by HX0039 can target and reduce the extracellular polysaccharides secreted by *Ralstonia solanacearum* (tobacco bacterial wilt pathogen) and weaken the antioxidant capacity of the pathway (67). Therefore, lipopeptides and various bioenzymes produced by strain HX0039 against pathogenic bacteria may constitute important mechanisms underlying the antifungal effects of this strain.

In recent years, with the development of and innovation in molecular biology, genomics technology has provided new avenues for the study of biocontrol mechanisms. Whole-genome sequencing is a comprehensive molecular biology technique. In this study, we integrated Oxford Nanopore Technology and next-generation sequencing to determine and analyze the whole-genome DNA sequences of organisms. Through sequence analysis and functional gene annotation (including the NR, KEGG, GO, and CARD databases), the mechanism underlying the strain phenotype can be elucidated at the gene level using anti-SMASH software (68). Lei et al. isolated the endophyte *Bacillus subtilis* G5, which can effectively inhibit *Magnaporthe oryzae*, from the interroot of rice, and 14 secondary metabolite synthesis gene clusters were predicted to encode antifungal substances, such as fengycin, surfactin, and bacilysin. To combat corn head smut caused by the fungus *Sporisorium reilianum*, a strain identified as *B. velezensis* 160 was applied in the field, where it decreased disease incidence and increased crop productivity (69). Comparative analysis of the genomes of four other *B. velezensis* strains revealed that they shared 2,804 genes and clusters for the production of difficidin, bacillibactin, bacilysin, macrolantin, bacillaene, fengycin, butirosin A, locillomycin, and surfactin (70). To elucidate the biocontrol mechanism of strain HX0039, we analyzed its genomic features and identified a repertoire of known and uncharacterized secondary metabolite BGCs (Table S3). These clusters are likely responsible for producing diverse microbial natural products, including structurally distinct compounds such as polyketides and NRPs, both of which are critically involved in antifungal biosynthesis (71, 72). Specifically, the genome encodes key biocontrol-related genes directing the synthesis of bioactive metabolites, such as fengycin, bacillaene, macrolactin H, surfactin, lichenysin, and bacillibactin, mediated by genes including *fenABCDE*, *yngI*, *yngG*, *bmyABC*, *ituABC*, *pks2ABCDEFG*, *srfAABC*, *lchAABC*, and *dhbABF* (Fig. 3 and Table S4).

Notably, our study focused on biocontrol genes associated with antifungal substances, particularly those hypothesized to play key roles in antagonizing “Sanghuang” green mold (*T. virens* SH4). Among these, iturin A emerged as a primary candidate: it was both predicted by anti-SMASH analysis and detected in co-cultures of HX0039 and SH4. This aligns with relevant reports: Wang et al. (73) demonstrated that iturin inhibits *Aspergillus niger* by disrupting membrane integrity, inducing oxidative stress, and perturbing glycolysis/gluconeogenesis and the tricarboxylic acid cycle. Similarly, C14-iturin A was identified as the major fungicidal factor in *Bacillus amyloliquefaciens* NCPSJ7, blocking conidial germination and damaging fungal cell walls and membranes; associated phenotypes (e.g., mitochondrial swelling, glycogen accumulation, autophagic bodies) further indicated impaired cellular function (74). In *B. amyloliquefaciens* S185, iturin A5 delays spore germination and disrupts polarity establishment in *Fusarium oxysporum* f. sp. *cubense*, highlighting its role as a key bioactive component (75). Consistent with these findings, our study observed that HX0039 disrupts the membrane integrity of *T. virens* SH4 hyphae (Fig. 4). Future studies are warranted to investigate the specific effects of iturin A on the cell wall integrity of *T. virens* SH4, further clarifying its antifungal mechanism.

### Conclusion

In conclusion, this study revealed that strain HX0039 has a potent biocontrol effect on “Sanghuang” green mold disease. Strain HX0039 can produce metabolites such as iturin A, fengycin, surfactin, macrolactin A, and bacillibactin, thereby exerting antifungal effects. Notably, compared with the HX0039 monoculture, the dual-culture group generated three characteristic peaks identified as iturin A, which suggests that iturin A may play a role in combating *T. virens* SH4. In addition, strain HX0039 has been proven to be reliable and safe for application. Taken together, these findings provide alternative strategies to control “Sanghuang” green mold disease caused by *Trichoderma* species.

## Data Availability

Data will be made available on request. For the 16S rRNA, *gyrA*, and *gyrB* genes of HX0039 strain, the GenBank accession numbers are OP268577, OR859838, and OR859839, respectively. For the circular chromosome and plasmid (pHX0039) sequences of HX0039 strain, the GenBank accession numbers are CP136263 and CP136264, respectively.

## SUPPLEMENTAL MATERIAL

Supplemental material aem.00005-25-s0001.docx Tables S1 to S4; Fig. S1 to S5.

ASM does not own the copyrights to Supplemental Material that may be linked to, or accessed through, an article. The authors have granted ASM a non-exclusive, world-wide license to publish the Supplemental Material files. Please contact the corresponding author directly for reuse.
