# Supplementary material for: Antagonistic mechanism of Bacillus velezensis HX0039 as a biocontrol agent against Trichoderma virens-induced “Sanghuang” green mold
Source: Appl Environ Microbiol. 2025 Jul 8;91(8):e00005-25. doi: 10.1128/aem.00005-25 (PMC12366312; doi:10.1128/aem.00005-25)
Supplement: Supplemental material — Tables S1 to S4; Fig. S1 to S5. [file aem.00005-25-s0001.docx]

**Supplementary Material**

**Antagonistic mechanism of *Bacillus velezensis* HX0039 as a biocontrol agent against *Trichoderma virens*-induced ‘Sanghuang’ green mold**

Ying Zhang^a,b,1^, Wenheng Gao^a,b,1^, Hui Zhang^a,b,1^, Tao Sun^a,c^, Huixiang Yang^a,b^, Yan Liu^d^, Xiaoyang Han^b^, Dengke Yin^a,b,*^, and Weifang Xu^a,b,^^[[1]](#footnote-1)^

^a^ Joint Research Center for Chinese Herbal Medicine of Anhui of IHM, Anhui University of Chinese Medicine, Hefei, 230012, China

^b^ Anhui Province Key Laboratory of Research and Development of Chinese Medicine, School of Pharmacy, Anhui University of Chinese Medicine, Hefei, 230012, China

^c^ College of Life Science and Technology, China Pharmaceutical University, Nanjing, 211100, China

^d^ Institute of Sericulture Science and Technology Research, Chongqing, 400700, China

**Table S1** Percentage of average nucleotide identity (ANI) between strain HX0039 and related *Bacillus* species.

| Strain | GenBank No. | ANI% | GC content/% | Size/bp |
| --- | --- | --- | --- | --- |
| *Bacillus velezensis* NJN-6 | CP007165 | 99.91 | 46.55 | 4,052,546 |
| *Bacillus velezensis* 19573-3 | CP067043 | 99.40 | 46.56 | 3,990,203 |
| *Bacillus velezensis* LF01 | CP058216 | 99.35 | 46.56 | 3,974,023 |
| *Bacillus velezensis* BY6 | CP051011 | 99.41 | 46.61 | 3,898,273 |
| *Bacillus velezensis* 160 | CP119675 | 99.43 | 45.85 | 4,296,610 |
| *Bacillus velezensis* QST713 | CP025079 | 97.64 | 45.9 | 4,233,757 |
| *Bacillus velezensis* SQR9 | CP006890 | 97.63 | 46.1 | 4,117,023 |
| *Bacillus tequilensis* EA-CB0015 | CP048852 | 77.54 | 43.74 | 4,012,371 |
| *Bacillus subtilis* BSD-2 | CP013654 | 77.07 | 43.88 | 4,030,837 |
| *Bacillus subtilis* 7PJ-16 | CP023409 | 77.37 | 43.28 | 4,209,045 |
| *Bacillus licheniformis* T5 | CP124852 | 72.59 | 46.16 | 4,247,430 |
| *Bacillus licheniformis* TAB7 | CP027789 | 72.67 | 45.82 | 4,367,367 |
| *Bacillus pumilus* SAFR-032 | NC009848 | 70.91 | 41.29 | 3,704,641 |
| *Bacillus megaterium* HGS7 | CP065213 | 68.65 | 38.27 | 5,035,031 |
| *Bacillus megaterium* WSH-002 | CP003017 | 68.75 | 38.24 | 4,983,975 |
| *Bacillus megaterium* QM B1551 | CP001983 | 68.65 | 38.26 | 5,097,129 |
| *Bacillus cereus* ATCC 14579 | NC004722 | 68.08 | 35.28 | 5,411,809 |
| *Bacillus cereus* AR156 | CP015589 | 68.27 | 35.48 | 5,160,326 |
| *Paenibacillus polymyxa* SQR-21 | CP006872 | 65.92 | 45.64 | 5,828,436 |

Note: Taxonomic study of the bacterial strain HX0039 based on the most closely related bacterial strains belonging to closely related *Bacillus* sp. after average nucleotide identity (ANI) analysis. Species delineation thresholds were set at ANI values of 95%, which are widely accepted in microbial taxonomy for distinguishing bacterial species.

**Table S2** General features of the strain HX0039 genome.

| Features | Chromosome | Plasmid |
| --- | --- | --- |
| Size(bp) | 4,073,512 | 63,977 |
| G + C content (%) | 46.43% | 41.84% |
| Number of CDSs | 3,921 | 81 |
| tRNA | 82 | 0 |
| rRNA | 27 | 0 |

Note: CDSs represents protein-coding sequences.

**Table S3** Overview of secondary metabolic gene clusters in the genome of *B. velezensis* HX0039 predicted by antiSMASH.

| Most similar known cluster | fengycin | bacillaene | macrolactin H | - | - | - | surfactin | locillomycin/locillomycin B/locillomycin C | bacilysin | bacillibactin | - | difficidin | - | - | - |
| --- | --- | --- | --- | --- | --- | --- | --- | --- | --- | --- | --- | --- | --- | --- | --- |
| Similarity Confidence | High | High | High |  |  |  | High | Low | High | High |  | High |  |  |  |
| To | 148,640 | 313,356 | 624,991 | 817,85 | 973,457 | 1,096,724 | 1,692,119 | 1,839,144 | 2,359,815 | 2,946,311 | 3,561,403 | 3,690,886 | 3,772,581 | 3,890,922 | 4,020,942 |
| From | 10,812 | 203,233 | 536,777 | 788,966 | 952,717 | 1,055,480 | 1,626,712 | 1,761,421 | 2,318,397 | 2,880,950 | 3,540,513 | 3,584,720 | 3,751,532 | 3,849,822 | 3,999,059 |
| Type | NRPS,betalactone | transAT-PKS,NRPS,NRPS-like,T3PKS | transAT-PKS | lanthipeptide-class-ii | terpene | PKS-like | NRPS | NRPS,transAT-PKS | other | iPP-like,NRP-metallophore,NRPS,terpene-precursor | terpene-precursor | transAT-PKS | terpene-precursor | T3PKS | terpene |
| Region | Region 1 | Region 2 | Region 3 | Region 4 | Region 5 | Region 6 | Region 7 | Region 8 | Region .9 | Region .10 | Region 11 | Region 12 | Region 13 | Region 14 | Region 15 |

Note: The symbol '-' indicates that no similar biosynthetic gene clusters (BGCs) were found. In this study, BGC similarity was determined by comparison against existing BGC entries in the "Minimum Information about a Biosynthetic Gene Cluster" (MIBiG) database.

NRPS = non-ribosomal peptide synthetase; PKS = polyketide synthase; transAT-PKS = trans-acyltransferase polyketide synthetases; T3PKS = type III polyketide.

**Table S4** Gene statistics of predicted secondary metabolites detected in the genome of HX0039.

| Metabolite | Gene name | | Length/bp | Strain name (NCBI GenBank) |
| --- | --- | --- | --- | --- |
| Fengycin | | *fenA* | 7,659 | *Bacillus velezensis* FZB42 ([CP000560.1](https://www.ncbi.nlm.nih.gov/nuccore/CP000560.1?from=1921412&to=1970953)) |
|  | | *fenB* | 7,698 |  |
|  | | *fenC* | 7,650 |  |
|  | | *fenD* | 10,776 |  |
|  | | *fenE* | 3,804 |  |
|  | | *yngG* | 900 |  |
|  | | *yngI* | 1,641 |  |
| Bacillomycin D | | *bmyA* | 11,949 | *Bacillus velezensis* FZB42 ([CP000560.1](https://www.ncbi.nlm.nih.gov/nuccore/CP000560.1?from=1921412&to=1970953)) |
|  | | *bmyB* | 16,092 |  |
|  | | *bmyC* | 7,860 |  |
| Iturin | | *ituA* | 11,939 | *Bacillus subtilis* (AB050629.1) |
|  | | *ituB* | 1,6089 |  |
|  | | *ituC* | 7,857 |  |
| Macrilactinh | | *pks2A* | 2,307 | *Bacillus velezensis* FZB42 (AJ634061.2) |
|  | | *pks2B* | 12,261 |  |
|  | | *pks2C* | 4,773 |  |
|  | | *pks2D* | 8,739 |  |
|  | | *pks2E* | 7,005 |  |
|  | | *pks2F* | 5,721 |  |
|  | | *pks2G* | 7,383 |  |
| Surfactin | | *srfAA* | 10,755 | *Bacillus velezensis* FZB42 (AJ575642.1) |
|  | | *srfAB* | 10,761 |  |
|  | | *srfAC* | 3,837 |  |
| Lichenysin | | *lchAA* | 10,743 | *Bacillus licheniformis* DSM 13 (AJ575642.1) |
|  | | *lchAB* | 10,767 |  |
|  | | *lch AC* | 3,849 |  |
| Bacillibactin | | *dhbA* | 786 | *Bacillus subtilis subsp. subtilis str.* 168 (AL009126.3) |
|  | | *dhbC* | 1,197 |  |
|  | | *dhbF* | 7,137 |  |

Note: The above genes originated from the Biosynthetic Gene Clusters (BGCs) located in regions 1, 3, 7, and 10 of antiSMASH prediction results.

**
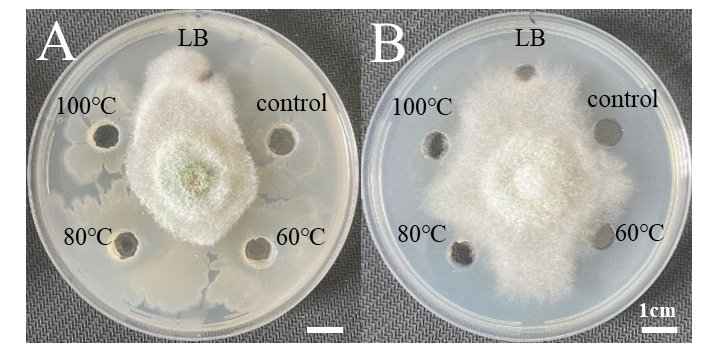
**

**FIG S1** Antagonistic effects against *T. virens* SH4 of heat-treated HX0039 cell suspension (A) and cell-free supernatant (B). The mycelial disc of SH4 (5 mm) was inoculated in the middle of the PDA plate.


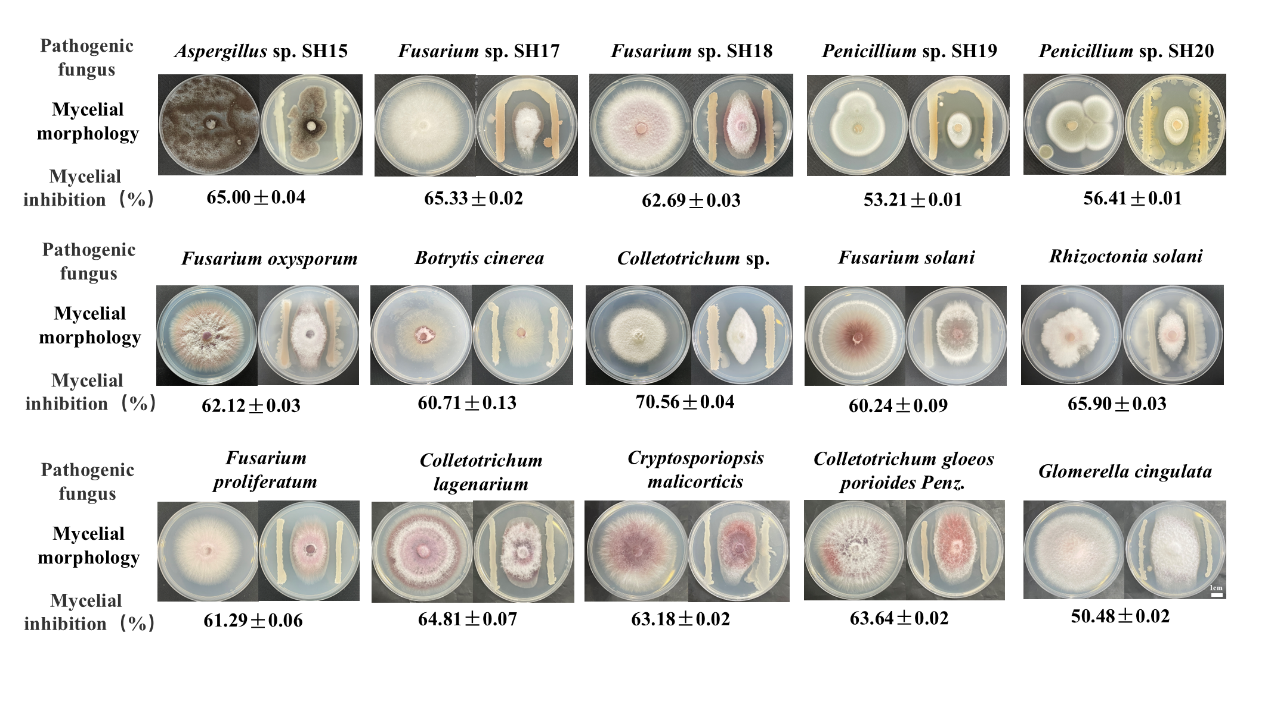
**FIG S2** Antifungal activity of strain HX0039 against the selected pathogenic fungi. The left panel shows the control group, where a mycelial disc of the pathogenic fungus was inoculated onto a standard Potato Dextrose Agar (PDA) plate. In the right panel, the pathogenic fungus was inoculated at the center of a PDA plate, with the HX0039 cell suspension (10⁸ CFU/mL) streaked on both sides. The mycelial inhibition rate (I) was calculated using the following formula: I (%) = [ (the growth diameter of the fungal pathogen in the control group - the growth diameter of the pathogen in the HX0039 treatment plate)/(the growth diameter of the fungal pathogen in the control group-the diameter of the tested fungal agar disc (5.0 mm) ] × 100


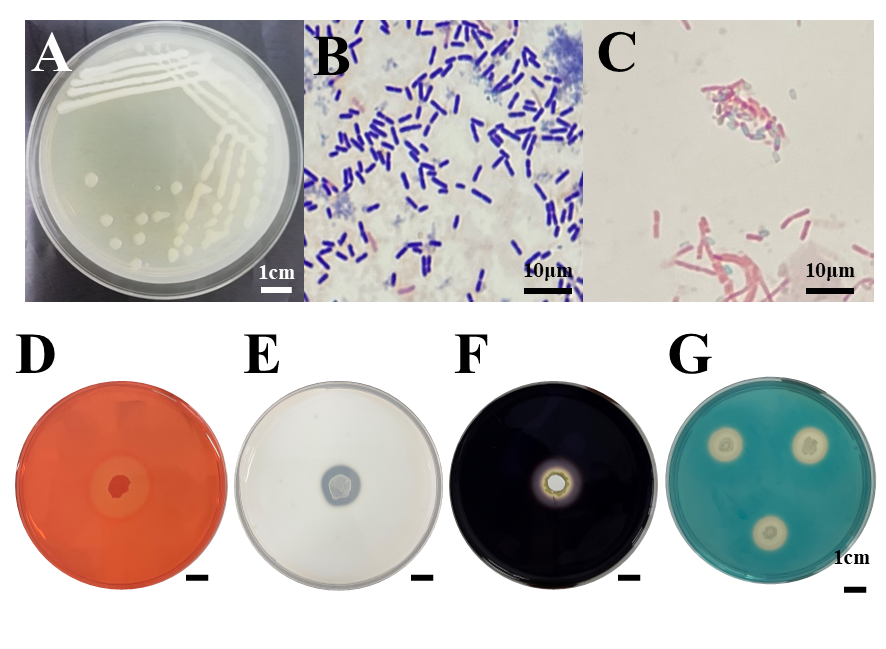


**FIG S3** Morphological characterization and enzyme activity detection for strain HX0039. (A) Colony characteristics of strain HX0039 on LB after 24 h. (B) Gram staining. (C) Spore staining. (D) Cellulase detection. (E) Protease detection. (F) Amylase detection. (G) CAS plate detection the bacillibactin of strain HX0039. Note: LB=Luria–Bertani liquid medium


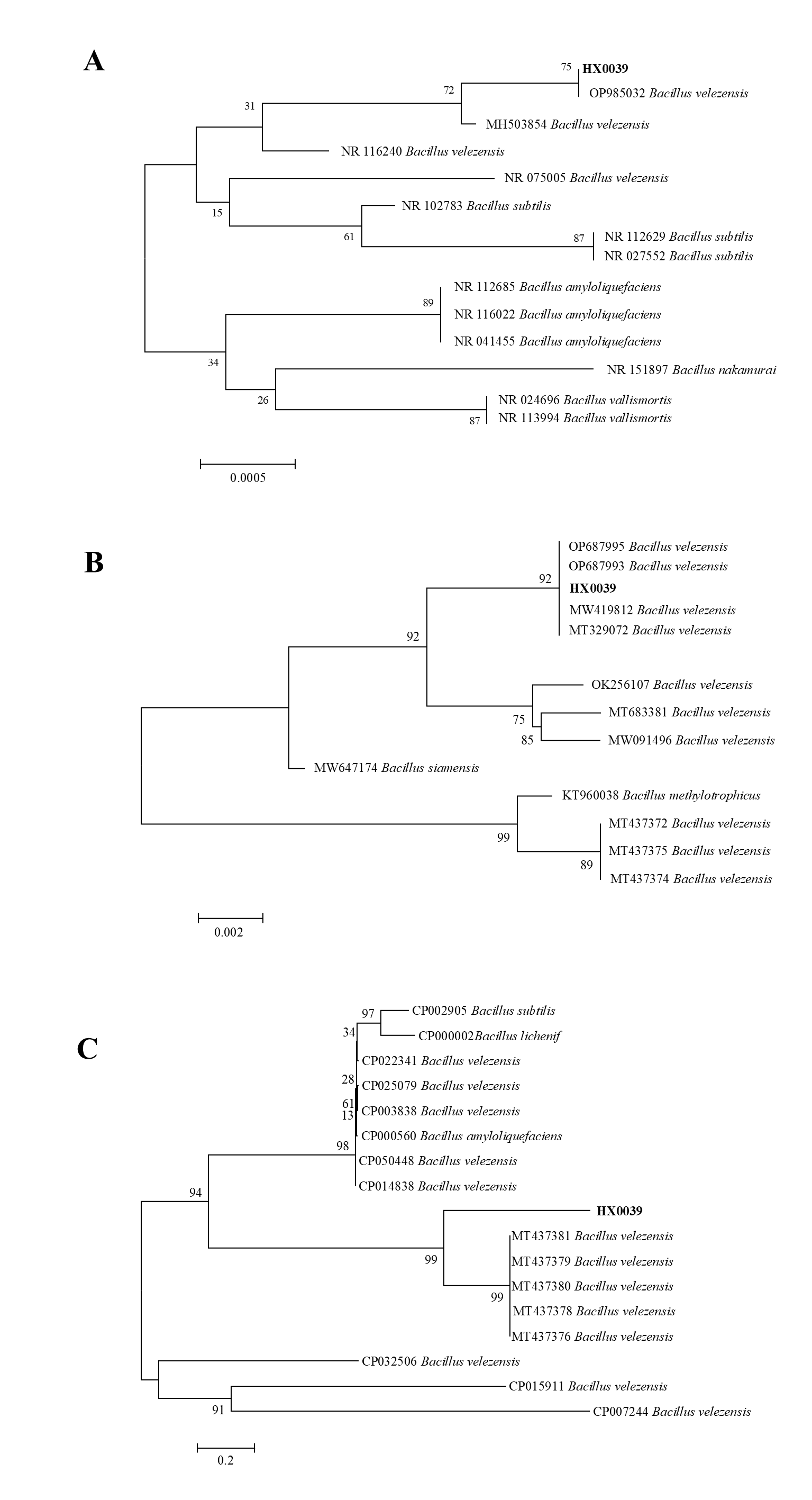


**FIG S4** Phylogenetic trees of strain HX0039 based on 16S rRNA (A), *gyrA* (B) and *gyrB* (C) gene sequences. Phylogenetic trees were constructed using the neighbor-joining method in MEGA software version 7.0. Three separate phylogenetic trees based on each of the above single housekeeping genes were generated, with bootstrap values of 1000 replicates included for each tree.


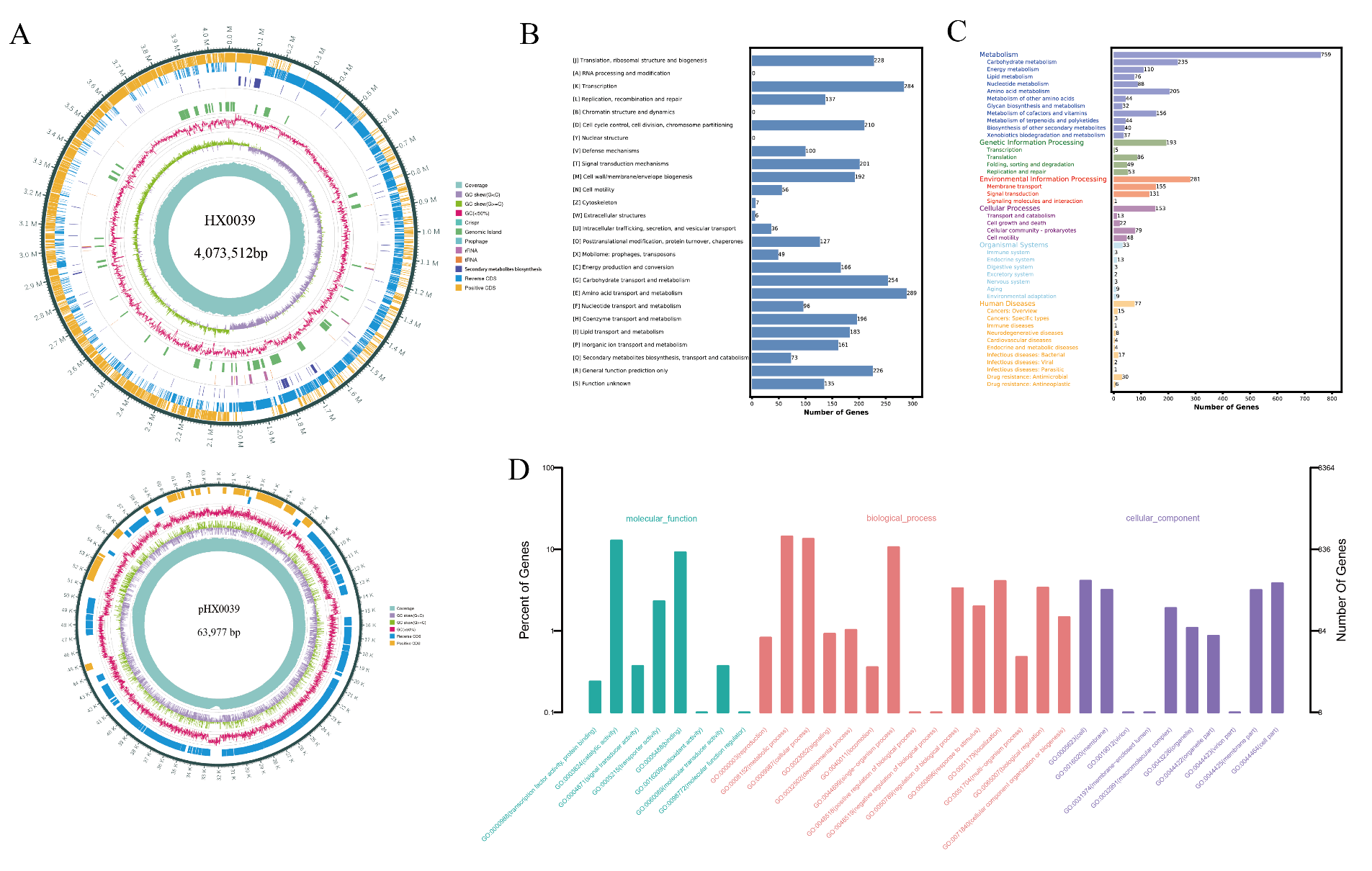


**FIG S5** Genome maps of *B. velezensis* HX0039 and genome function annotation. (A) Circular maps of the chromosome and plasmid. The genomic circular diagrams from the outside to inside show the following: coding genes (righteous strand), coding genes (negative-sense strand), secondary metabolite gene density, tRNA (orange) and rRNA (purple), CRISPR, prophage and gene islands, GC ratio, GC skew, sequencing depth, plasmid circle map coding gene (righteous strand), coding gene (negative-sense strand), GC ratio, GC skew, sequencing depth. (B) Genome-encoded protein COG functional classification statistics. (C) KEGG functional classification statistics of genome-encoded proteins. (D) GO functional classification statistics of genome-encoded proteins.

1. Corresponding authors.

   E-mail addresses: yindengke@ahtcm.edu.cn (D. Yin), xuweifang@ahtcm.edu.cn (W. Xu)

   ^1^These authors contributed equally to this work. [↑](#footnote-ref-1)
